# The Science for Profit Model—How and why corporations influence science and the use of science in policy and practice

**DOI:** 10.1371/journal.pone.0253272

**Published:** 2021-06-23

**Authors:** Tess Legg, Jenny Hatchard, Anna B. Gilmore

**Affiliations:** Department for Health, Tobacco Control Research Group, University of Bath, Bath, United Kingdom; University of Calfornia San Francisco, UNITED STATES

## Abstract

Science has been at the centre of attempts by major industries, including tobacco, chemical, and pharmaceutical, to delay progress in tackling threats to human and planetary health by, *inter alia*, obscuring industry harms, and opposing regulation. Some aspects of this influence are well documented, others remain poorly understood, and similarities between industries remain underexplored. This study, therefore, aims to synthesise the literature to develop an evidence-based typology and model of corporate influence on science in order to provide an overview of this multi-faceted phenomenon. We obtained literature examining corporate attempts to influence science and the use of science in policy and practice from: database searches, bibliographies, expert recommendations, and web alerts; using a modified scoping review methodology (n = 68). Through interpretive analysis we developed the Science for Profit Typology and Model. We identified eight corporate sectors repeatedly engaging in activities to influence science, including: manipulation of scientific methods; reshaping of criteria for establishing scientific “proof”; threats against scientists; and clandestine promotion of policy reforms that increase reliance on industry evidence. The typology identifies five macro-level strategies used consistently across the eight industries, comprising 19 meso-level strategies. The model shows how these strategies work to maximise the volume, credibility, reach, and use of industry-favourable science, while minimising these same aspects of industry-unfavourable science. This creates doubt about harms of industry products/practices or efficacy of policies affecting industry; promotes industry-favoured policy responses and industry products as solutions; and legitimises industry’s role as scientific stakeholder. These efforts ultimately serve to weaken policy, prevent litigation, and maximise use of industry products/practices—maximising corporate profitability. We provide an accessible way to understand how and why corporations influence science, demonstrate the need for collective solutions, and discuss changes needed to ensure science works in the public interest.

## Introduction

Science has been at the centre of attempts by corporate sectors including the tobacco, chemical, and fossil fuels industries to delay progress in tackling threats to human and planetary health [1–3]. Those who have studied corporate involvement in science suggest it is undertaken “not simply out of benevolence or an altruistic desire to support scientific inquiry” [4], but to purposefully-create misinformation, doubt, and ignorance (or “agnogenesis”)–to, *inter alia*, obscure the harms of industry products and practices, and oppose environmental, occupational and public health regulation that could threaten corporate profits [2, 5, 6]. Whilst industry funded science is growing [7, 8], so too is sector-specific evidence that “information strategies” involving the use of science represent a key mechanism of corporate political influence [9–11].

Recognising that different industries often use similar approaches to exert political power [10, 12, 13], frameworks for understanding corporate power and influence across multiple industries have been called for [10, 14, 15]. Researchers have begun to develop such frameworks, but some earlier works did not explicitly identify science as a key tenet [16, 17]. Emerging models of the commercial determinants of health [18–20] identify science as an important route of influence, but none of these, nor the sector-specific taxonomies of corporate political activity [9–11], examine or categorise science in detail.

There is, therefore, a pressing need to comprehensively map corporate influence on science. Such an overview can inform decision-making concerning whether collective science-based solutions are justified, and if so, which might be most impactful. In 2010, White and Bero began this cross-industry categorisation process, analysing documents—mostly made available through litigation—from five industry sectors (tobacco, pharmaceutical, vinyl chloride, lead and silicosis-generating industries), developing six high-level categories of science manipulation [3]. Additional evidence identifying both further aspects of scientific influence [21–23] and the use of similar strategies by other industry sectors [24–26] indicates that corporate influence on science is even more complex, multifaceted, and widespread. However, despite the growing evidence base showing wider-ranging strategies and synergies between industry approaches, there has been little attempt to collate and make sense of the literature on corporate influence on science in its entirety.

To date, overviews of the literature that investigate multiple industries have offered rich narrative syntheses of industries’ scientific strategies [1, 2, 27]; synthesised literature on discrete parts of this influence on science, such as on research agendas [28]; and mapped relationships through which health research may be affected by corporate interests [29]. However, no scholarly work has systematically categorised industry strategies to give a comprehensive yet detailed picture of the entire phenomenon. Further, while existing overviews document the longstanding evidence on industries such as tobacco and pharmaceuticals, knowledge concerning sectors such as alcohol [30], gambling [25] and mining [31] often remains absent, since this evidence has only more recently emerged.

To address this knowledge gap, this study aimed to synthesise the large and methodologically diverse literature on industry attempts to influence science, to develop an evidence-based typology and model of corporate influence on science and use of science in policy and practice. We used an unpublished rapid scoping review, “The Use of Science in Policy” (Ulucanlar, 2015) and White and Bero’s 2010 paper [3] to develop the following research questions:

- Which industries attempt to influence science and its use in policy and practice?
- What strategies do these industries use to influence science and its use in policy and practice, and for what intended purpose?
- Are there similarities in the strategies used by different industries (and their intended impacts) that would enable the creation of collective solutions?

Our typology and model build on previous research by expanding the breadth of industries investigated (inductively identifying corporate sectors from the literature), expanding the breadth and depth with which industry strategies are examined (including by identifying strategies not included in previous cross-sector analyses), and illustrating the desired effects and outcomes of these corporate scientific strategies.

## Methods

To obtain literature investigating corporate influence on science and its use in policy and practice we conducted a modified scoping review based on a procedure proposed by Arksey and O’Malley [32] and subsequently refined [33, 34]. Scoping reviews are useful when mapping methodologically and substantively diverse literature, especially where theoretical frameworks have not been established, and we followed the five proposed stages with some modifications and additions.

In line with Arksey and O’Malley’s advice that parameters for areas of particular focus in a scoping review are set once a sense of the “volume and general scope of the field has been gained” [32], pilot electronic database searches were undertaken. These enabled us to set the focus on corporate influence on science for policy and practice, and to exclude literature which solely investigated corporate influence on science for product development or marketing, thus ensuring the study size remained feasible. With the assistance of a subject librarian, a two-phase search strategy was then developed. First, In July 2017, through electronic database searches on Web of Science, Scopus and PubMed) using the search criteria (corporat* or industr*) AND (influence or tactic or strategy) AND (science or evidence) AND review we obtained peer-reviewed papers summarising knowledge of corporate influence on science and its use in policy and practice. Next, between July 2017 and June 2020, we obtained additional literature that contained further information on industry sectors or strategies from:

- bibliographies of included literature
- expert recommendations (we contacted researchers expert in the field of corporate influence on science specific to each of the industries identified in the stage one searches—what Arksey and O’Malley call “existing networks” [32]—and requested recommendations of key texts); and
- web alerts (automated searches for new literature using the same search string as the database searches, except the requirement for literature to be a review).

**Fig 1:**
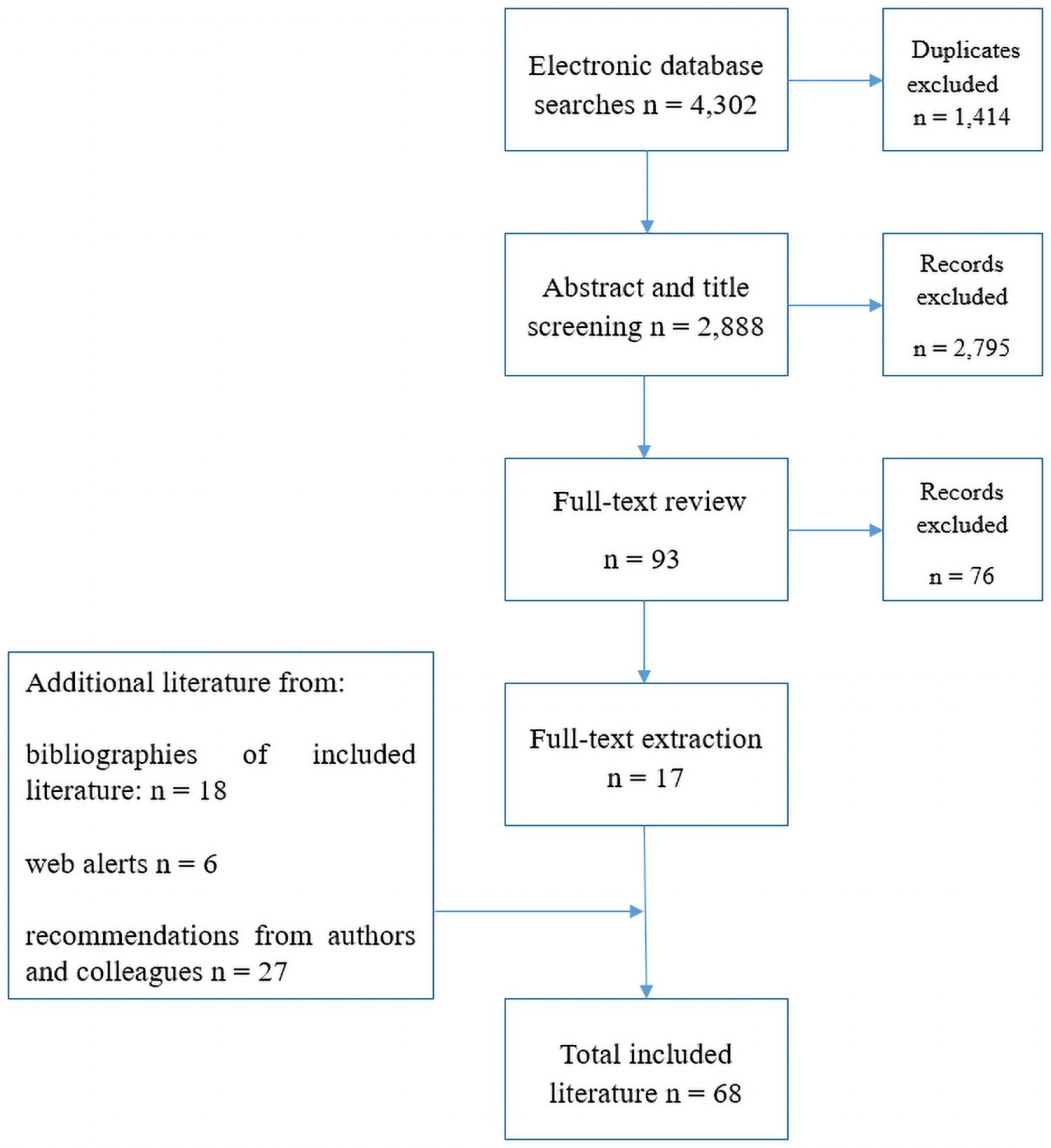
Study selection process.

See Box 1 for full inclusion and exclusion criteria for literature sampled and Fig 1 for the study selection process.

**Box 1:** Inclusion and exclusion criteria.

Criteria for all literature obtained
Inclusion: Written in English Investigates strategies used by industry to influence science or the use of science in policy and practice Exclusion: Investigates corporate influence on science which is solely for product development or marketing No restrictions on: Geographical location investigated Literature whose authors declared potential conflicts of interest^1^
Criteria specific to literature obtained in stage one^2^ searches
Inclusion: Peer reviewed Published in or after 2010 (subsequent to White and Bero’s paper) (3) Reviews a section of the evidence base (e.g. systematic reviews/qualitative syntheses but also literature which synthesises knowledge without explicitly stating its methods)
Criteria specific to literature obtained in stage two^2^ searches
Inclusion: Contains additional knowledge to that within the literature already sampled (i.e. the aim was for data saturation concerning knowledge on corporate influence on science, rather than to achieve an exhaustive sampling of all available literature) No restrictions on: Peer-reviewed status (reports, book chapters and so on also included) Publication date Study design (primary studies also included)

^1^ Five studies were included where authors declared funding from, or a conflict of interest in relation to, the industry/industries investigated in the study. Once the typology was finalised, this subset of studies was re-analysed separately (using framework matrices in NVivo 12). No substantive differences were found between industry strategies documented here and those within the main sample, and these studies are included in the final sample.

^2^ Stage one = electronic database searches; Stage two = bibliographies of other included literature, expert recommendations, and web alerts.

Stage two literature searches were conducted concurrently with data extraction and analysis—that is, we worked iteratively—where knowledge gaps were identified in the developing typology, literature was purposively sampled to fill them. This is an addition to traditional scoping review methodologies, where gaps are not targeted as the focus of subsequent rounds of data collection. Most of the study selection process was conducted by TL, however all authors screened the small number of papers where there was uncertainty about inclusion.

Subsequently, in an extension to Arksey and O’Malley’s method (which recommends reporting findings from each study consecutively), we interpretively analysed the literature to develop a typology and model. In NVivo 12, we created a six-point list of industry strategies (inputted as ‘nodes’) using White and Bero’s labels [3]–“fund”, “publish”, “suppress”, “distort public discourse”, “set standards” and “disseminate.”—as *a priori* initial categories. We uploaded pdfs of the eligible studies and coded them line-by-line, identifying strategies used by industries to influence science and/or the use of science in policy and practice, and assigning each piece of data to one of our nodes. Where new industry strategies were identified, we expanded and added to the nodes (including by creating new codes and micro codes within them).

TL conducted the initial coding, AG second-coded twenty per cent of the papers (which needed particularly careful analysis). We grouped the different ways industries attempt to influence science and its use of science in policy and practice into 5 macro-level strategies (broken down into 19 meso-level and 64 micro-level strategies), and coded the intended effects and outcomes of the strategies, where this was made explicit in the literature.

All authors met monthly to discuss the literature, examine areas of complexity, review coding decisions, and develop the typology and model. We sought to ensure the rigour of our interpretation through the use of “prolonged engagement” (ensuring sufficient time is spent with the data) and “persistent observation” (ensuring a focus on the complexities within the data) [35]. As the typology and model developed, further informal discussions were held with colleagues expert in this research area.

## Findings

Based on our inclusion and exclusion criteria, we included 68 pieces of literature: 17 from electronic database searches, 18 from hand searching bibliographies, 27 from expert recommendations, and 6 from web alerts. See S1 Appendix for details of included literature.

### Corporate sectors and strategies

Within this literature, we identified eight corporate sectors (alcohol; chemicals and manufacturing; extractive; food and drink; fossil fuels; gambling; pharmaceuticals and medical technologies; and tobacco) engaging in activities to influence science and/or its use in policy and practice. These sectors contained diverse, often multi-national, corporations. For example, within the food and drink industry, evidence related to corporations manufacturing products including confectionary, sugar-sweetened beverages, breakfast cereals, meat, infant formula, food additives and dietary supplements. A detailed breakdown of industry sectors, number of included studies investigating each sector, and sectors excluded due to insufficient data is provided in S2 Appendix.

We found corporations engaged in **five macro strategies** to influence science and the use of science in policy and practice. These macro strategies in turn comprise 19 meso strategies and 64 micro strategies. All strategies—macro, meso and micro—shown in the Science for Profit Typology (Table 1), are mutually reinforcing and often used in conjunction with each other. The macro and meso strategies are outlined briefly in the following section, while a fuller account of each including their purposes, key examples of the micro-level detail and supporting evidence is provided in S3 Appendix.

**Table 1:** The Science for Profit Typology—Macro, meso and micro strategies used by industry to influence science and the use of science in policy and practice.

Macro strategies	Meso strategies	Micro strategies
**A. Influence the conduct and publication of science to skew evidence bases in industry’s favour**	1. Fund and undertake “safe” research	1.1 Fund and undertake “safe” research which distracts attention from industry harms, frames industry and industry products as part of the ‘solution’, and promotes interventions that minimise damage to product sales
		1.2 Commission lawyers and public relations firms to manage research programmes to ensure research is “safe”
		1.3 Fund and undertake research to identify or demonstrate public perceptions
	2. Covertly undertake or prevent “risky” industry research	2.1 Covertly undertake “risky” research so that it can be hidden or abandoned
		2.2 Prevent “risky” industry research from being undertaken
	3. Control design & analysis of industry-funded science to ensure favourable results	3.1 Control the design and analysis of industry-funded primary studies
		3.2 Control the design and analysis of industry-funded evidence syntheses
	4. Shape and undermine external research	4.1 Shape external (e.g. governmental) organisations’ research priorities through access, funding, and political power
		4.2 Attempt to block the funding of potentially unfavourable independent research
		4.3 Deliberately obstruct independent data collection
	5. Ensure favourable research is heavily represented in the evidence base	5.1 Maximise the presence of industry-funded publications in the peer-reviewed literature
		5.2 Fund or create journals to have influence over what is published
		5.3 Create publications which emulate peer-reviewed/quality science
	6. Control reporting and suppress publication of unfavourable science	6.1 Control the way in which unfavourable industry science is reported within publications
		6.2 Suppress publication of unfavourable science
**B. Influence the interpretation of science to undermine unfavourable science and create a distorted picture of the evidence base**	7. Develop and promote criteria and concepts for critiquing science which can be used to further industry arguments	7.1 Develop criteria for the conduct and interpretation of science (including determining scientific “proof”) with the intention that these can be used to undermine unfavourable science
		7.2 Adopt the concepts of “junk science” and “sound science” for use in undermining unfavourable science and promoting industry science
		7.3 Fund and coordinate public relations campaigns to promote these industry-friendly criteria and concepts to key stakeholders
	8. Obtain and re-analyse raw data from unfavourable science	8.1 Obtain raw data underlying unfavourable research by enabling data access legislation, and pressuring or litigating against scientists
		8.2 Re-analyse independent data including that acquired in these ways to refute unfavourable findings
	9. Attack and misrepresent science	9.1 Attack the methods of unfavourable science (tailoring the criteria depending on the nature of the science to be attacked)
		9.2 Label unfavourable science “junk”
		9.3 Misrepresent single pieces of evidence
		9.4 Misrepresent whole evidence bases
		9.5 Misrepresent expert consensus
	10. Monitor and attack scientists and organisations	10.1 Monitor the opposition in order to weaken it
		10.2 Attack individual scientists and whole cohorts of researchers
		10.3 Remove individual scientists from positions of power
		10.4 Attack organisations that create and disseminate science
**C. Influence the reach of science to create an “echo chamber” for industry’s scientific messaging**	11. Use legal means to protect industry evidence from being discovered or accessed	11.1 Prevent research from being undertaken in countries where corporations are vulnerable to litigation based on legal advice
		11.2 Limit internal communication to mask industry knowledge of harms based on legal advice
		11.3 Store scientific documents in ways which would prevent their discovery based on legal advice
		11.4 Use “proprietary information” claims when pressed by courts to release industry evidence
		11.5 Attempt to embed mechanisms in trade and investment treaties which prevent access to industry evidence
		11.6 Silence plaintiffs using secret payments
	12. Contract messengers to create scientific “echo chambers”	12.1 Create front groups to amplify industry-friendly scientific messages
		12.2 Fund third parties in order to amplify or shape their scientific stances
		12.3 Recruit, fund, and train individuals to be trusted scientific voices for industry
		12.4 Strategically create and fund a multitude of voices to manufacture a picture of scientific consensus
		12.5 Build industry coalitions (both within an industry, and with allied industries) to demonstrate support for industry-friendly scientific stances
	13. Fund, produce and disseminate materials which package science in industry-favourable ways	13.1 Fund, produce and disseminate lobbying materials that summarise science in industry-favourable ways
		13.2 Fund, produce and disseminate textbooks, letters to the editor, practice guidelines and other educational or academic materials
		13.3 Fund, produce and disseminate “easily digestible” materials such as factsheets, newsletters, and product information materials
		13.4 Obtain and disseminate reprints of industry-favourable scientific publications
	14. Use education, events, and meetings to disseminate industry-favourable scientific messages to key stakeholders	14.1 Fund, organise and speak at “educational events” for key stakeholders
		14.2 Access policymakers through meetings and hearings
		14.3 Infiltrate decision-making contexts in order to ensure industry-friendly scientific stances are heard
		14.4 Train and use industry representatives to meet with health professionals
	15. Maximise press coverage of industry-favourable scientific messages	15.1 Create favourable media content
		15.2 Fund media outlets in order to influence what is disseminated
		15.3 Co-opt journalists through media training and conference funding
		15.4 Design events intended to maximise media coverage
		15.5 Demand media coverage of industry scientific arguments in the name of “balance”
**D. Create industry-friendly policymaking environments which shape the use of science in policy decision-making in industry’s favour**	16. Implement and utilise industry-friendly standards of evidence in regulatory decision-making	16.1 Attempt to implement industry-friendly standards of evidence which set a high evidential bar within regulatory decision-making
		16.2 Utilise such standards in attempts to undermine the use of unfavourable science in regulatory decision-making
	17. Secure and utilise policymaking reforms which increase reliance on and provide a conduit for industry-favourable evidence	17.1 Secure the implementation and use of mandatory regulatory tools such as business impact assessments which increase reliance on industry data and prioritise evidence on economic impacts
		17.2 Secure and utilise changes to upstream policymaking architecture which embed industry’s right to participate in policymaking processes
		17.3 Secure reductions in evidence requirements for some regulatory decision-making to maximise the use of industry-favourable evidence
**E. Manufacture trust in industry and its scientific messaging**	18. Manufacture a picture of industry credibility	18.1 Ensure and normalise industry’s presence in academic settings in attempts to gain trust and scientific credibility within academia
		18.2 Promote industry’s overt links with expert individuals and organisations to manufacture a picture of industry credibility more broadly
		18.3 Inflate the credibility of industry science, scientists, and scientific perspectives
	19. Conceal industry’s involvement in science, scientific messaging and influence on policy reforms that affect the use of science	19.1 Conceal industry funding of science, production of science and recruitment of scientists
		19.2 Conceal industry dissemination of science and scientific messages
		19.3 Conceal industry attempts to shape ways in which science is used in policymaking

See S3 Appendix for fuller details on these strategies including purposes, supporting evidence and examples of micro-level detail.

We observed remarkable consistency across the eight industries in their use of the macro strategies, with all industry sectors using Macro Strategies A to D, and five of the eight industries using Macro Strategy E. There was greater diversity in the use of the 19 meso strategies, ranging from 10 out of the 19 used by the gambling industry, up to all 19 used by the tobacco industry (Table 2).

**Table 2:** Macro and meso strategies used by each sector of industry.

Macro Strategies A-E and meso strategies 1–19 (grey cells indicate where we did not identify attempts to influence science, not evidence of lack of industry activity)	Industry sectors							
	Alcohol	Chemicals and manufacturing	Extractive	Food and drink	Fossil fuels	Gambling	Pharma and medical tech	Tobacco
**A. Influence the conduct and publication of science to skew evidence bases in industry’s favour**	✔□	✔□	✔□	✔□	✔□	✔□	✔□	✔□
1. Fund and undertake “safe” research	✔□	✔□	✔□	✔□	✔□	✔□	✔□	✔□
2. Covertly undertake or prevent potentially “risky” industry research		✔□			✔□		✔□	✔□
3. Control design and analysis of industry-funded science to ensure favourable results	✔□	✔□	✔□	✔□	✔□		✔□	✔□
4. Shape and undermine external research			✔□	✔□		✔□		✔□
5. Ensure favourable research is heavily represented in the evidence base	✔□	✔□	✔□	✔□	✔□		✔□	✔□
6. Control reporting and suppress publication of unfavourable science	✔□	✔□	✔□	✔□	✔□	✔□	✔□	✔□
**B. Influence the interpretation of science to undermine unfavourable science and create a distorted picture of the evidence base**	✔□	✔□	✔□	✔□	✔□	✔□	✔□	✔□
7. Develop and promote industry-friendly criteria and concepts for critiquing science		✔□			✔□			✔□
8. Obtain and reanalyse raw data from unfavourable science		✔□		✔□	✔□		✔□	✔□
9. Attack and misrepresent science	✔□	✔□	✔□	✔□	✔□	✔□	✔□	✔□
10. Monitor and attack scientists and organisations	✔□	✔□		✔□	✔□	✔□	✔□	✔□
**C. Influence the reach of science to create an ‘echo chamber’ for industry’s scientific messaging**	✔□	✔□	✔□	✔□	✔□	✔□	✔□	✔□
11. Use legal means to protect industry evidence from being discovered or accessed		✔□	✔□				✔□	✔□
12. Contract messengers to create scientific “echo chambers”	✔□	✔□	✔□	✔□	✔□	✔□	✔□	✔□
13. Fund, produce and disseminate materials which package science in industry-favourable ways	✔□	✔□	✔□	✔□	✔□	✔□	✔□	✔□
14. Use education, events, and meetings to disseminate industry-favourable scientific messages to key stakeholders	✔□	✔□	✔□	✔□	✔□	✔□	✔□	✔□
15. Maximise press coverage of industry-favourable scientific messages	✔□	✔□	✔□	✔□	✔□	✔□	✔□	✔□
**D. Create industry-friendly policymaking environments which shape the use of science in policy decision-making in industry’s favour**		✔□		✔□	✔□		✔□	✔□
16. Implement and utilise industry-friendly standards of evidence in regulatory decision-making		✔□		✔□	✔□			✔□
17. Secure and utilise policymaking reforms which increase reliance on and provide a conduit for industry-favourable evidence		✔□			✔□		✔□	✔□
**E. Manufacture trust in industry and its scientific messaging**	✔□	✔□	✔□	✔□	✔□	✔□	✔□	✔□
18. Manufacture a picture of industry credibility	**✔□**	**✔□**		**✔□**	**✔□**	**✔□**	**✔□**	**✔□**
19. Conceal industry’s involvement in science, scientific messaging and influence on policy reforms that affect the use of science	**✔□**	**✔□**	**✔□**	**✔□**	**✔□**		**✔□**	**✔□**
**Total macro (out of 5**) & meso (out of 19) strategies	**4** (12)	**5** (18)	**4** (12)	**5** (15)	**5** (17)	**4** (10)	**5** (16)	**5** (19)

### Macro Strategy A—Influence the conduct and publication of science to skew evidence bases in industry’s favour

Corporate influence on the conduct and publication of science is often an attempt to pre-empt or refute independent science which paints corporations or their products unfavourably. The purpose, therefore, is to effectively counter and outweigh this unfavourable science. To achieve this, corporations operationalise six meso strategies **(Strategies 1–6)**, which influence what research is, and is not, undertaken and published. Over time, this influence works to skew the evidence base in favour of industry through two routes. First, evidence deemed unfavourable—that which implicates industry in harm or casts doubt on the efficacy or necessity of industry products and practices, or promotes interventions or policies that could damage industry profits—is minimised. Second, evidence deemed favourable–that which obscures industry harms, argues for alternative causes of harm (including through allocating blame to individuals rather than corporations), promotes supposed benefits of industry products and practices, or casts doubt on interventions and policies that could damage profits–is maximised.

Corporations fund and undertake research that, whilst potentially being methodologically sound, is guaranteed to produce “safe” outcomes for industry because of its focus **(Strategy 1)**. For example, when independent research began to show the link between cancer and smoking, the tobacco industry funded its own research on other potential causes of cancer (including hormones and “nervous tension”) to distract attention from the independent evidence that threatened the future of the industry [27]. Such research is also used to promote industry products as beneficial to public health, even when simpler, cheaper solutions may exist, such as food industry-funded research focusing on specific nutrients rather than whole foods [28]. Corporations also use this “safe” research to promote industry-favoured interventions (rather than mandatory regulation of industry), such as alcohol industry-funded research on alcohol education programmes [36]. Such research promotes the idea that individual-level public health measures are favourable, and enables industry, through engagement in science, to signal it is responsibly working to minimise the harms caused by its products.

While publicly undertaking this “safe” research, “risky” industry research is undertaken secretly so it can be hidden or abandoned if results prove unfavourable **(Strategy 2)**. For example, on realising its research on nicotine addictiveness in rats was not obtaining the desired results, tobacco corporation Philip Morris ordered the lead scientist to “close down his laboratory, to kill the animals, to suspend all further investigation… never try to publish or discuss his work… and to find work elsewhere” [2].

Industries also control the design and analysis of research (both primary studies and research syntheses) to ensure it is favourable to industry **(Strategy 3)**. In Papua New Guinea, a mining company systematically underestimated the harms caused by its mine, by failing to measure certain variables and ignoring background levels of chemicals in their analyses [31]. Corporations have also cherry-picked papers for inclusion in evidence syntheses, such as an American Plastics Council-funded review on bisphenol A (a plastic in products such as food cans) that found no toxicity at low levels, but was later criticised for only including a minority of available studies [37].

Industries use access, funding, and political power in attempts to shape and undermine the research conducted by *external* organisations (that is, organisations set up independently from industry, such as public research bodies) **(Strategy 4)**. For example, the sugar industry gained access to the expert panel of the US National Institute of Dental Research as a means of shaping the US government’s research priorities towards interventions that did not involve restricting the consumption of sugar [38].

Industries also work to ensure favourable research is heavily represented in the evidence base **(Strategy 5)**. As far back as the 1930s, the silica industry was funding the *Journal of Industrial Hygiene and Toxicology*, which in return agreed to publish the industry’s own abstracts [3]. Industries have also artificially inflated the prevalence of their research in peer-reviewed publications by repeatedly publishing the same research, such as a trial on the efficacy of antipsychotic drug, Risperdal, which was published in six different journals using different author names [1]. In addition to prolifically publishing peer-reviewed works, industries seek to create publications which emulate such literature, for example by holding symposia in order to enable publication (and subsequent citation) of (non-peer-reviewed) proceedings that favour industry products [39].

When research conducted *publicly* proves unfavourable to industry, strategies are used to control access to it **(Strategy 6)**, such as the pharmaceutical industry threatening legal action against researchers who attempt to publish unfavourable results [1], and the gambling industry requiring scientists to sign legal agreements waiving their rights to publication [25].

### Macro Strategy B—Influence the interpretation of science to undermine unfavourable science and create a distorted picture of the evidence base

Corporations attempt to influence the interpretation of science through four meso strategies **(Strategies 7–10)**. These work collectively to minimise the perceived credibility of unfavourable science and maximise the perceived credibility of favourable science by distorting how science, scientists, and scientific organisations are seen by the public and experts alike.

In response to unfavourable evidence demonstrating the harms of passive smoking, the tobacco industry promoted criteria and concepts (developed from existing guidelines created by the Chemical Manufacturers Association) for critiquing science **(Strategy 7)**. One part of this was to call for a rejection of evidence that failed to show a relative risk greater than two (i.e. that did not indicate that the industry product/practice in question at least doubled the risk of harm) [23]. With approximate relative risks of lung cancer and heart disease from second-hand smoke of 1.2 and 1.3 respectively, this strategy was intended to prevent policy action which would protect the public from second-hand smoke [23]. These criteria were promoted though the “Sound Science” and “Good Epidemiology” industry-run public relations campaigns, which demanded unrealistic and, at times, unobtainable levels of evidence in epidemiological studies examining harms caused by industry products. The intention was to create doubt about the evidence base [23, 40].

Another industry strategy is to obtain and re-analyse raw data from unfavourable science in order to undermine it **(Strategy 8)**. This has been operationalised by pressuring and litigating against researchers, and working to implement data access legislation (which enabled industry to access data from publicly-funded science) [39]. For example, when a researcher found that children were seen to have positive reactions to RJ Reynolds’ mascot, “Joe Camel”, the tobacco company litigated and gained access to the researcher’s data (including lab books and participants’ personal information) [2]. Once data such as this is obtained it can be reanalysed. For instance, data from a study which had found an association between beryllium (a metal used in weapons manufacture) and lung cancer was reanalysed by industry-funded scientists at a product defence firm (that had also worked extensively with the tobacco industry). By changing some parameters in the analysis, the “elevation of lung cancer rates was no longer statistically significant” and the beryllium industry used this revised research to counter independent evidence showing harms [37].

Attacking and misrepresenting the evidence base is a way to challenge unfavourable science that implicates industry products or practices as causes of harm **(Strategy 9)**. For example, The Society of the Plastics Industry attacked an article in *The Lancet*, which reported an increased foetal death rate among the wives of men who had been exposed to vinyl chloride monomer (VCM), a gas used in the production of plastic, calling the paper “worthless”, “naïve” and “misleading” [3].

At first glance, attacks on the methods of unfavourable science can appear to be legitimate, scientific arguments. However, they are often based on industry-friendly criteria (such as those created in Strategy 7) and are used in contradictory ways, depending on the context. For example, Egilman and Billings argue that “when a suspect carcinogen is found to cause cancer in human epidemiologic studies but not in animals, companies argue that animal studies are required to prove causation. On the other hand, when animal studies are positive and human epidemiology is incomplete or negative, companies argue that human evidence is required before the government can regulate the substance and before workers and others can be compensated” [41].

Attacks on scientists and scientific bodies **(Strategy 10)** are used to weaken any opposition. When independent scientists researched the harms caused to children from lead exposure, the lead industry labelled one academic an “over-emotional, untrustworthy anti-lead fanatic” [1].

### Macro Strategy C—Influence the reach of science to create an “echo chamber” for industry’s scientific messaging

Having worked to create a skewed evidence base (through Macro Strategy A) and skewed interpretations of the evidence base (through Macro Strategy B), industries work to maximise the reach of their preferred scientific messages (Macro Strategy C). This macro strategy is operationalised by five meso strategies **(Strategies 11–15)**, which work together to maximise the reach of favourable science and scientific messages (including negative messaging about independent science) and to minimise access to (and therefore reach of) unfavourable industry science. Ultimately, this create an “echo chamber” effect, whereby industries’ own favourable science and its messaging about others’ science is widely disseminated and amplified, whilst industry’s unfavourable evidence is hidden.

Industries use legal means to protect their own unfavourable science from being discovered or accessed **(Strategy 11)**. Lawyers advised British American Tobacco not to conduct research in certain countries such as Canada, Germany, and Brazil, since they were seen as “places… helpful to plaintiffs” [42]. CropLife America and the European Crop Protection Association (lobbying groups representing pesticides corporations) worked to embed “exclusive use periods” and “confidential business information” provisions in a trade and investment treaty between the United States and the European Union (EU). Although treaty negotiations were stalled, these mechanisms would have had the effect of blocking public access to industry data on product risks [43].

Corporations contract a multitude of messengers–“friendly” voices—to amplify scientific messages and distance these messages from industry **(Strategy 12)**. Messengers include front groups created by industry, third-party organisations (including thinktanks, professional associations, and PR firms), “expert” individuals, and allied industries. For example, the Beverage Institute for Health and Wellness (BIHW) was created by Coca-Cola “to present its version of facts about nutrition…to health professionals. Because position statements come from BIHW, not Coca-Cola itself, they appear to be issued by a legitimate unbiased source of scientific information” [44]. Trusted individuals including scientists, health professionals, and police officers are also used to disseminate industry-friendly scientific messages, such as the alcohol industry funding dieticians to promote the health benefits of beer [29]. As part of British American Tobacco’s efforts to secure policy reforms in the EU that would shape the use of science in policy decision-making in industry’s favour, it created a broad coalition of corporations including from the pharmaceutical, chemicals, and fossil fuels industries–a “policy network” through which to claim widely held support for the reforms [21].

Industries also fund, produce and disseminate materials that package science in industry-favourable ways **(Strategy 13)**. Such “packaged science” (often created through third parties, obscuring industry involvement) includes: lobbying materials (such as policy submissions); educational or academic materials (such as textbooks and practice guidelines); “easily digestible” materials (such as factsheets); and reprints of industry-favourable science. For example, the industry-funded International Centre for Alcohol Policies (ICAP) created much “packaged science”, such as the controversial “Drinking in Context: Patterns, Interventions and Partnerships” output, which was disseminated to policymakers in low- and middle-income countries for use as a policy brief, but has been “widely criticised for misrepresenting the public health view on alcohol policies” [45].

Corporations use education, meetings, and events to maximise stakeholder exposure to industry-friendly scientific messages **(Strategy 14)**. The pharmaceutical industry sponsors continuing medical education (CME), its “substantial influence” over the content providing an opportunity to control the scientific messages that reach health professionals [46]. Industry also uses events to reach policymakers. For example, in the 1960s, the head of the Lead Industry Association (LIA) appeared at a subcommittee on pollution, saying “lead causes no public health hazard in America today… [on] the basis of vast clinical evidence… the general public is not now, nor in the immediate future, facing a lead hazard” [3]. Previous to this event, the LIA had suppressed unfavourable research which did not support their public stance [3]. In addition to simply meeting with decision-makers, industries also work to infiltrate decision-making bodies to ensure industry-friendly scientific messages are heard. This can result in the blurring of lines between industry and independent bodies. One example is The American National Standards Institute, a public body in the United States [3]. Two prominent members of its Committee on Toxic Dusts and Gases were employees of the LIA, and in their official roles at the Institute campaigned to lower the safety standards for levels of lead permitted in the atmosphere of work places [3].

Industries also work aggressively to ensure the presence of industry-friendly scientific messages in the media (**Strategy 15**). In one instance, the chemicals industry hired PR company Hill and Knowlton, which helped the major tobacco companies create doubt about the harms of smoking [47], in order to organise a campaign of “maximum media exposure” on the science of dioxin (a by-product of industrial practices) [2]. The company trained scientists to communicate industry-friendly stances on dioxin, and organised over 400 media interviews in five months, helping to establish the industry as an “authority on dioxin research” [2]. Media messaging is also controlled though industry funding of media outlets, such as the fossil fuels industry-funded Tech Central Station, described as “part of a corporate PR machine that helps corporations like ExxonMobil… get their message out” [24].

### Macro Strategy D—Create industry-friendly policymaking environments which shape the use of science in policy decision-making in industry’s favour

The strategies already described (Macro strategies A-C) work collectively to ensure a bias towards the use of industry-favourable scientific evidence in policy and practice. However, industries have gone beyond this and attempted (in some cases successfully) to create industry-friendly policymaking environments which shape the use of science in policy decision-making in industry’s favour (macro strategy D). This macro strategy is operationalised by two meso strategies **(Strategies 16–17)** through which industries have attempted to embed standards of evidence in policymaking, and embed other policy reforms that increase reliance on, and provide a conduit for, industry-favourable evidence. Together, these two strategies work to maximise the use of favourable science in policymaking, and minimise the use of unfavourable science in policymaking, ultimately making it harder to pass regulation that threatens corporate profits.

Corporations have attempted to establish and embed the use of industry-friendly scientific standards into regulatory decision-making (as part of a risk-based rather than precautionary-based approach to policymaking), which aim to set the evidential bar high enough to dismiss unfavourable evidence and thus prevent regulation **(Strategy 16)**. For example, the tobacco industry lobbied in the 1990s to shape risk assessment of industry products, intending to embed rules into EU regulatory mechanisms for how epidemiological and animal data should be assessed [23, 48]. Such efforts, although ultimately unsuccessful, would have mandated the use of criteria for determining scientific “proof” which were drawn up by industry themselves and required a relative risk of harm greater than two to be established before a product could be regulated (see Strategy 7) [23, 48].

Pesticides lobby groups CropLife America and the European Crop Protection Association have also pushed for a risk-based approach to regulation, using trade and investment treaty negotiations (which later stalled). They attempted to embed a toxicological risk assessment approach to the evaluation of endocrine-disrupting chemicals, aiming to lower standards of protection against pesticides in the EU to US levels [43]. This was despite experts concluding that, due to limits on current research methodologies, it would be nearly impossible to accurately determine a safe level of exposure to endocrine disruptors, which have been shown to break “all the rules and assumptions that have guided toxicology through the era of modern chemical regulation” [43]. Experts argue that this risk-based approach to regulation, what industry lobby groups have labelled a “science-based” approach, is ultimately intended to prevent precautionary approaches to policymaking, since it means regulators must wait for definitive evidence that a product is dangerous (an often unobtainable goal) before they are able to regulate it [49].

The tobacco industry has had more success embedding industry-friendly scientific standards in US policymaking environments, however, where Philip Morris was the driving force behind the implementation of the US Information Quality Act (IQA—also known as the Data Quality Act). This Act required government agencies to produce quality guidelines for the science used in their decision-making, rather than relying on peer-review as a quality standard, as was previously the case. It also embedded a mechanism through which third parties (including industry) could challenge the science used, on the basis of these quality standards (often in ways outlined in strategy 9) [2]. In the five years after its implementation, the IQA was used by the chemicals, food and fossil fuels industries to contest and dismantle evidence bases unfavourable to their interests by challenging minor aspects of many individual studies which had collectively contributed to “weight-of-the-evidence risk judgements” [2].

Corporations have also secured and utilised policymaking reforms that increase reliance on and provide a conduit for industry-favourable science **(Strategy 17)**. British American Tobacco (along with other sectors of industry including chemicals and fossil fuels) promoted a set of regulatory reforms in the EU, now known as “Better Regulation” or “Smart Regulation” because they thought these reforms would make it harder to pass public health policies which countered their interests [21, 22, 50]. One strand of this reform was to promote the use of business impact assessments which took a cost benefit analysis approach, in which impacts to business are effectively prioritised over other impacts such as to health or the environment [51, 52]. Another strand was the mandatory use of stakeholder consultation which, while ostensibly about transparency and good governance [50], in fact mandated industry’s right to be heard early in scientific debates about their products and practices [52]. This process has been described as “an opportunity for highly resourced corporations to slow, weaken, or prevent public health policies” [53]. Both the tobacco and chemicals industries have gone on to use these requirements to promote their own misleading evidence and undermine public health evidence [21, 43, 53, 54].

### Macro Strategy E—Manufacture trust in industry and its scientific messaging

Finally, industries use two strategies to underpin and enable all the preceding macro strategies (A-D) and meso strategies (1–17). They do so either by manufacturing a picture of industry credibility **(Strategy 18)** or concealing industry involvement in science, which works to enhance the credibility of both industry’s science and its messaging on how science should be interpreted and used **(Strategy19)**.

To frame industry as an appropriate and even essential partner in science and scientific decision-making, and to create legitimacy around industry and their science, corporations manufacture trust in themselves **(Strategy 18)**. They do so by funding academics (e.g. through grants, honoraria, awards and consulting fees), students (e.g. via generous studentships), and academic infrastructure, thereby normalising a corporate presence in academia and creating dependence on industry. For example, the Department of Plant and Microbial Biology at the University of California Berkeley received $50 million from Novartis (a Swiss pharmaceutical corporation). Critics argued that the funding (which was for research and laboratory equipment) would limit the academic freedom of the university and result in a “marked change in the very soul of Berkeley” [55].

Industry promotes such funding and the links with academia it creates as evidence “it is seeking the ‘truth’ about the dangers of its products” [2] and to manufacture a picture of credibility more broadly (i.e. beyond academia). For example, ExxonMobil contributed to the $225m Global Climate and Energy Project at Stanford University, and then went on to advertise its relationship with the “best minds”, speaking of “lively debate” about greenhouse gases and climate change [2]. When tobacco giant Philip Morris created its Worldwide Scientific Affairs Programme, one criteria for deciding whether to fund a research application was “whether it would enhance the credibility of the company” [39].

At times, industries work overtly with organisations outside university settings to boost their credibility. Examples include Coca-Cola becoming corporate partner to professional body The American Dietetic Association [56], and the gambling industry making voluntary contributions to the funding body, The Responsible Gambling Trust.

Conversely, and notably where their credibility is already damaged, industries also attempt to obscure the provenance of their science and scientific messages to increase the perceived legitimacy of such **(Strategy 19)**. The tobacco industry has used many third parties to hide its influence on science and the use of science, such as the Center for Indoor Air Research (used to fund favourable projects on “indoor air pollution”) [27], the law firm Covington and Burling (used to covertly recruit scientists who would go on to create research refuting the effects of passive smoking) [57], and the European Policy Centre (which concealed British American Tobacco’s attempts to influence upstream policymaking in the EU, in turn influencing the way in which science was used) [51].

These two underpinning strategies–manufacturing a picture of industry credibility and concealing industry involvement—may at first glance seem contradictory. However, these strategies illustrate the adaptable and multi-faceted nature of corporate influence on science. Corporations sometimes use one strategy, sometimes the other, and sometimes both, enabling them to either *promote* industry’s role in the creation, reach, interpretation, and use of science, or *hide* exactly these links, depending on what would serve the industry’s goals in each specific context.

## Discussion

### Summary

This paper is the first to attempt to fully categorise corporate influence on both science and the use of science in policy and practice. It shows that corporate influence on science goes far beyond a handful of industry actors working nefariously to skew isolated evidence bases. Instead, it involves industries permeating and moulding scientific, academic, and policymaking systems to ensure such systems work in their interest. We identified 5 macro, 19 meso, and 64 micro strategies through which this influence is enacted (the Science for Profit Typology—Table 1), finding that these strategies are used consistently and repeatedly by diverse corporations across eight industry sectors–alcohol, chemicals and manufacturing, extractive, food and drink, fossil fuels, gambling, pharmaceuticals and medical technologies, and tobacco. All eight industries were seen to use either four or all five of the macro strategies, and between 10 and 19 of the 19 meso-level strategies (Table 2).

**Fig 2:**
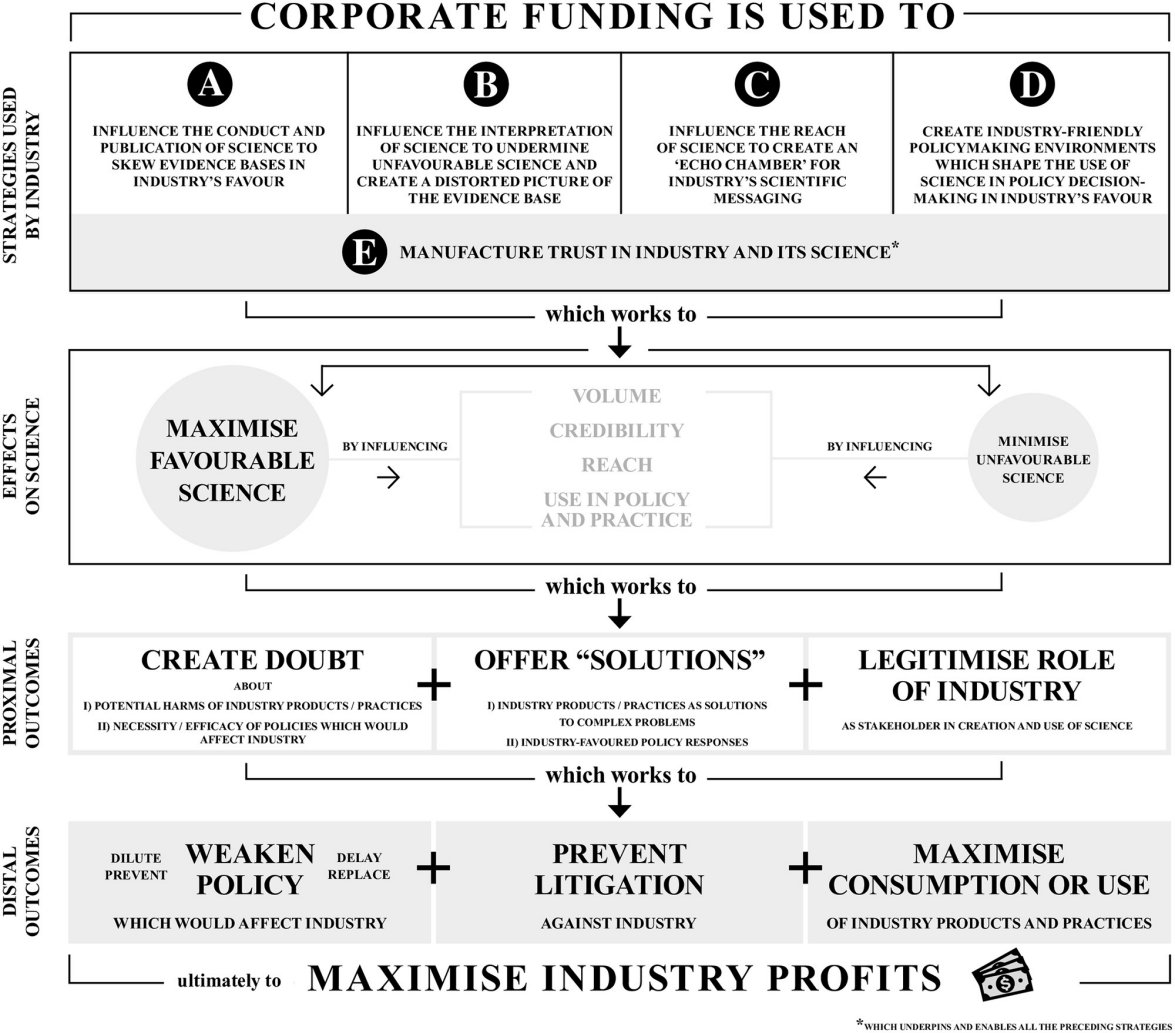
The Science for Profit Model—Corporate influence on science and the use of science in policy and practice—Strategies, effects, and outcomes.

Our analysis also demonstrated these industries’ attempts to influence science were undertaken for similar reasons. We developed a model of corporate influence on science and the use of science in policy and practice (the Science for Profit Model–Fig 2) to illustrate the mechanisms through which this influence is mediated, identifying four stages of influence, which we name “strategies”, “effects on science”, “proximal outcomes” and “distal outcomes”.

The model shows that the corporate macro, meso and micro strategies identified work collectively to achieve the following effects on science–to maximise the volume, credibility, reach, and use of industry-favourable science, and to minimise those same aspects of industry-unfavourable science. In this way, corporations reshape and skew whole evidence bases in their interests (such that when, for example, researchers, policymakers and practitioners look to the literature for answers to problems they may, often unknowingly, find evidence bases flooded with research tailored for industry’s benefit), control who evidence does and does not reach (with evidence on industry harms sometimes not seeing the light of day while a plethora of trusted voices are used to ensure industry-funded misinformation reaches key audiences), and ultimately influence how evidence gets used.

These complex and multifaceted interactions with science lead to three proximal outcomes: they (i) create doubt about the potential harms of industry products and/or practices and about policies that might reduce product sales or profitability; (ii) promote industry-favoured policy responses and industry products as solutions to complex problems; and (iii) legitimise the role of corporations as stakeholders in science and, through this, society. These proximal outcomes in turn serve to create three distal outcomes–to weaken policy, prevent litigation, and maximise consumption and use of industry products and practices, ultimately maximising corporate profitability.

Perhaps most worrying was not just the scale and consistency of this corporate influence but that it extends substantially beyond influencing the production, credibility, and reach of science to shaping how science is used in policy and practice with potentially far-reaching societal impacts. For instance, we found that corporations have worked to promote an over simplistic risk-based, rather than precautionary-based approach to regulation, which sets a high evidential bar and enables industry to dismantle whole evidence bases paper by paper [2, 23, 48]. Industry has misleadingly referred to this as a ‘science-based’ approach despite the fact that it is specifically intended to make it harder for policymakers to both use whole evidence bases in what is a genuinely scientific approach, and to regulate corporate products [49]. To date, corporations have sought to implement such systems via both regulatory reforms and trade and investment treaties [43, 48], policy fora where public health and environmental interest groups may not routinely be present. Similarly, industries have worked to embed systems which, through the use of stakeholder consultations and impact assessments requiring a cost benefit approach, increase reliance on and provide a conduit for industry-favourable science [50–52]. Evidence from the EU shows that this was specifically intended to make it harder to pass policies that would protect human and planetary health and has gone on to be used in this way [21, 43, 51, 54]. Worryingly, such systems are widespread [58–62] and may be having adverse impacts on policymaking in many jurisdictions.

### Strengths and weaknesses

A key strength of our approach is that it creates an evidence-based, pan-industry typology and model. The typology provides an accessible way of understanding the diverse corporate strategies used and the intentions behind them, while the model outlines, simply, the ways in which these strategies lead to outcomes.

The particular strengths of our analysis over existing attempts to synthesise the evidence in this area are three-fold. First, it identifies industry sectors inductively, leading to the inclusion of several sectors that thus far had either been excluded from (the gambling industry) [27, 28, 63] or relatively neglected in (the alcohol [2, 27, 63], extractive [27, 29], food and drink [2, 27, 63] and fossil fuels [28, 29, 63] industries) this work. Second, it extends previous syntheses that focused on corporate influence on science by also examining the ways in which corporations have attempted to influence the use of science in policymaking. Thus, much of Macro Strategy D had not been outlined in previous syntheses. The sub-section of literature on which this strategy is based identifies a little recognised route of corporate influence [21], yet one which is vital to understand given its potential far reaching impacts. Third, we have provided significant detail on the industry strategies identified. Similar to previous work on corporate political activity that synthesised large volumes of evidence into evidence-based typologies [10–12], we identified and categorised corporate strategies not only broadly (the macro and meso strategies) but also in detail (micro strategies). This structured and hierarchical approach can aid in identifying key points in the system where change may be needed.

Where our data did not identify a specific industry’s use of a strategy, this should not be interpreted as evidence that industry does not use that strategy. This is particularly the case for strategies that are more covert and are therefore less likely to be documented. Examples include meso strategies 2 (covertly undertake or prevent “risky” industry research) and 11 (use legal means to protect industry evidence from being discovered or accessed); both evidenced in four of the eight industries. Further, analysis of corporate documents released following litigation against some industries has enabled the identification of more diverse strategies in these industries (the tobacco industry was found to use all 19 meso-strategies, the chemicals and manufacturing industry 18, and the pharmaceutical industry 16), when compared to industries where lawsuits have not provided such documents and where evidence is only now beginning to emerge (for example the gambling and extractive industries were found to use 10 and 12 of the 19, respectively).

We identified small amounts of data on other sectors including the banking [27] and tanning [64] industries but there was insufficient detail to include these in our analysis. There was also evidence that the tobacco industry planned to mobilise other industries such as the fishing and waterworks industries as part of their attempts to influence the use of science in policymaking [3, 65]. See S2 Appendix for information on sectors excluded due to lack of data. Finally, while there is evidence that the pharmaceutical, tobacco, fossil fuels, and chemicals and manufacturing industries have worked to influence the use of science in tort litigation through the Daubert Ruling in the United States [2, 40], the use of science in courts was beyond the remit of our study.

### Implications for policy and practice

The Science for Profit Typology can be used as an analytic framework for further research on corporate scientific activity. For example, it could be used to ascertain whether and how industry strategies change over time and place, and in response to policy interventions (that is, do industries diversify their scientific strategies when their activity is restricted in some areas), or to examine the strategies used by additional industries, extending the typology where appropriate. This has been done in a similar fashion with the initial evidence-based typologies of corporate policy influence strategies [10–12], which have then been used to investigate the corporate political activity of other industries [9, 66–68].

Our typology and the strategies therein intersect closely with the policy influence strategies identified in these corporate political activity (CPA) frameworks, in large part because the scientific strategies we identify are necessary building blocks in corporate influence. For instance, a key part of CPA, “information management” [11] identifies the production and use of misleading evidence as a key policy influence strategy. Our typology provides greater detail of the mechanisms through which this occurs. Another feature of CPA, “reputation management” [11] is also further elucidated in our typology in relation to science. Strategy 18 (manufacturing a picture of industry credibility) identifies how industries use interactions with science and academia to build their credibility, since this strategy functions not only to underpin and enable Macro Strategies A-D, but also to afford corporations greater credibility generally.

In short, influence on science does not occur in a vacuum; rather it is key part of the system through which corporate influence and power more generally are mediated. Amalgamating our typology with other models, overviews and conceptualisations of CPA and the commercial determinants of health (CDoH) [10, 11, 16–20, 69] could therefore provide a more holistic overview of how interactions with science serve corporate interests well beyond scientific and academic environments. This also indicates that addressing corporate influence on science is key to addressing CPA and CDoH more broadly.

Our typology and model can also inform future work in the field of agnotology [6]. Whilst not all agnogenesis (purposefully created ignorance) is created by corporations, or achieved through interactions with science, much is. As such, our work can further elucidate the relationship between corporate science-based strategies and the creation of agnogenesis.

### Identifying solutions to corporate influence on science

The key use of our work, however, is that the identification of industry strategies can be used to identify solutions. Our finding that scientific influence is widespread and enacted in similar ways and for similar reasons across diverse industries, indicates that collective solutions are both necessary and feasible.

The model and typology effectively identify two broad routes to achieve such solutions. The first to address the strategies identified one by one; the second to address the underlying driver–corporate funding of science. To date, many policies and practices have been proposed, developed, and utilised that mitigate the effects of the corporate strategies we identify. While it is beyond the scope of this paper to detail all of these, we outline some important ways to address each of the five macro strategies.

Attempts to tackle bias in the conduct and publication of science (Macro Strategy A) involve research integrity tools, used to assess risk of bias and improve reporting in science [70, 71]; mandatory registration of clinical trials [72]; and policies mandating reductions in author conflicts of interest [73] and prohibiting the publication of industry-funded science [74]. However, many such scientific protections can and have been disregarded, manipulated, and circumvented by corporate interests, and are therefore insufficient. For instance, it is often impossible to detect the influence that a corporate funder may have had on the design and execution of science [2]; journal policies that preclude the publication of industry-funded science can be circumvented through non-disclosure [63]; and corporations find alternative mechanisms for publication of their research, including through the creation of industry-funded journals and through publication of non-peer-reviewed proceedings from industry-funded symposia [1, 27].

Methods that have been suggested to mitigate corporate influence on the interpretation and reach of science (Macro Strategies B and C) include discontinuing industry-sponsored medical education [75], training consumers of science (including the public, journalists and health professionals) in evidence appraisal skills [76–78], and preventing industry relationships with civil society organisations [79].

Addressing industry efforts to shape the use of science in policy decision-making (Macro Strategy D) is complex. While the aim should be to prevent the further spread of such industry-friendly policymaking environments, a first step is to raise awareness among the public and policymakers of these strategies and how industry has used them to date, including to undermine policy action on endocrine-disrupting chemicals [40], carcinogenic solvents [2], tobacco [53, 54], and climate change [2].

Given its underpinning role, addressing industry attempts to manufacture trust in itself and its science (Macro strategy E), will be key. That industry uses its involvement in research to enable it to be seen as socially conscious and as a necessary partner in the search for solutions, often to problems it has created, is not yet typically understood as a key facet of “reputation management” [11]. As with many of the strategies identified, training in corporate influence on science as a key element of research training is an essential first step. Efforts to tackle concealment of industry involvement in science (that is, the second part of Macro strategy E) such as an author-centric database of researchers’ financial interests [80, 81] should be developed and implemented. However, since research has shown that declarations of conflicts of interest can have unanticipated impacts [82]; transparency measures are not a panacea.

Ultimately, however, addressing the underlying driver of much of this corporate influence on science is best achieved via structural changes to the way science is funded. A model for how corporate monies can be used to fund independent science has been elaborated for tobacco–essentially by mandating payments from industry which are then independently administered [83]. Such systems have been implemented in Italy, California, and Thailand, where levies on the pharmaceutical, tobacco, and alcohol industries have been used to fund independent research on their products [75, 84, 85]. Such an approach would help address all the other strategies identified and therefore likely represents the most effective and sustainable solution.

## Supporting information

S1 Appendix Included literature. (DOCX)

S2 Appendix Sectors of industry investigated. (DOCX)

S3 Appendix Macro, meso, and micro strategies used by industry to influence science and the use of science in policy and practice—Purposes, features, and examples. (DOCX)
