# Supplementary material for: The Science for Profit Model—How and why corporations influence science and the use of science in policy and practice
Source: PLoS One. 2021 Jun 23;16(6):e0253272. doi: 10.1371/journal.pone.0253272 (PMC8221522; doi:10.1371/journal.pone.0253272)
Supplement: S1 Appendix — (DOCX) [file pone.0253272.s001.docx]

**S1** **Appendix. Included literature.**

| Study | Sector(s) of Industry investigated | Search Stage identified | Overview or Primary Study | How it defines its Methods | Declares any funding from or conflict of interest related to the industry/ies investigated? |
| --- | --- | --- | --- | --- | --- |
| Studies identified from electronic database searches | | | | | |
| Andermann et al., 2016. Evidence for Health II: Overcoming barriers to using evidence in policy and practice | Tobacco  Pharmaceuticals and medical technologies | Electronic database search | Overview | ‘review’ | No |
| Babor & Robaina, 2013. Public health, academic medicine, and the alcohol industry's corporate social responsibility activities | Alcohol | Electronic database search | Overview | ‘analytic essay’ | No |
| Capps, 2016. Can a good tree bring forth evil fruit? The funding of medical research by industry | Tobacco  Pharmaceuticals and medical technologies  Food and drink  Alcohol | Electronic database search | Overview | ‘Critical analysis’ and ‘review’ | No |
| Cullerton et al., 2016. Playing the policy game: a review of the barriers to and enablers of nutrition policy change | Food and drink | Electronic database search | Overview | ‘Interpretive synthesis’ | No |
| Gasparyan et al., 2013. Conflicts of interest in biomedical publications: considerations for authors, peer reviewers, and editors. | Pharmaceuticals and medical technologies | Electronic database search | Overview | ‘This article overviews evidence on common instances of conflict of interest in research publications’ | No |
| Grundy et al., 2013. Interactions between Non-Physician Clinicians and Industry: A Systematic Review | Pharmaceuticals and medical technologies  Food and drink | Electronic database search | Overview | ‘systematic review’ | No |
| Moodie et al., 2013 Profits and pandemics: prevention of harmful effects of tobacco, alcohol, and ultra-processed food and drink industries | Tobacco  Alcohol  Food and drink | Electronic database search | Overview | No categorisation | Yes (one author declares previous research and consultancy fees, lecture fees, and travel fees from the pharmaceutical and food and drink industries. A second author declares relationships with organisations that have received alcohol industry funds). |
| Pinto, 2017. To know or better not to: Agnotology and the social construction of ignorance in commercially driven research | Tobacco  Fossil fuels  Pharmaceuticals and medical technologies | Electronic database search | Overview | No categorisation | No |
| Savell et al., 2014. How does the tobacco industry attempt to influence marketing regulations? A systematic review | Tobacco | Electronic database search | Overview | ‘systematic review’ | No |
| Savell et al. 2015. How does the alcohol industry attempt to influence marketing regulations? A systematic review | Alcohol | Electronic database search | Overview | ‘systematic review’ | No |
| Schott et al., 2010a. The Financing of Drug Trials by Pharmaceutical Companies and Its Consequences Part 1: A Qualitative, Systematic Review of the Literature on Possible Influences on the Findings, Protocols, and Quality of Drug Trials | Pharmaceuticals and medical technologies | Electronic database search | Overview | ‘qualitative systematic review’ | No |
| Schott et al., 2010b. The Financing of Drug Trials by Pharmaceutical Companies and Its Consequences Part 2: A Qualitative, Systematic Review of the Literature on Possible Influences on Authorship, Access to Trial Data, and Trial Registration and Publication | Pharmaceuticals and medical technologies | Electronic database search | Overview | ‘qualitative systematic review’ | No |
| Shimazawa & Ikeda, 2014. Conflicts of interest in psychiatry: Strategies to cultivate literacy in daily practice | Pharmaceuticals and medical technologies | Electronic database search | Overview | No categorisation | No |
| Smith et al., 2010a. Is the increasing policy use of Impact Assessment in Europe likely to undermine efforts to achieve healthy public policy? | Tobacco  Chemicals and manufacturing  Fossil fuels | Electronic database search | Overview | ‘this essay is a thematic analysis of literature’ | No |
| Smith et al., 2013. What is known about tobacco industry efforts to influence tobacco tax? A systematic review of empirical studies | Tobacco | Electronic database search | Overview | ‘systematic review’ | No |
| Spurling et al., 2010 Information from pharmaceutical companies and the quality, quantity and cost of physicians’ prescribing: a systematic review | Pharmaceuticals and medical technologies | Electronic database search | Overview | ‘systematic review’ | Yes (one author declares a previous financial relationship with a pharmaceutical corporation manufacturing generic drugs). |
| Stamatakis, 2013. Undue industry influences that distort healthcare research, strategy, expenditure and practice: a review | Pharmaceuticals and medical technologies | Electronic database search | Overview | review | No |
| Studies identified from bibliographies of other included studies | | | | | |
| Abraham, 2002 The pharmaceutical industry as a political player | Pharmaceuticals and medical technologies | Reference chained (from Akl, 2019) | Overview | No categorisation | No |
| Baba et al., 2005. Legislating “sound science”: the role of the tobacco industry | Tobacco | Reference chained (from Bero, 2013) | Primary study | No categorisation (documents analysis) | No |
| Bingham et al., 2003 Daubert: The most influential supreme court ruling you’ve never heard of | Tobacco  Chemicals and manufacturing  Fossil fuels  Food and drink  Pharmaceuticals and medical technologies | Reference chained (from Michaels and Monforton, 2005) | Report | Report | No |
| Brownell and Warner, 2009. The Perils of Ignoring History: Big Tobacco Played Dirty and Millions Died. How Similar is Big Food? | Tobacco  Food and drink | Reference chained (from Thomas et al., 2018) | Primary study | ‘A review and analysis of empirical and historical evidence’ | No |
| Diethelm and McKee, 2009. Denialism: what is it and how should scientists respond? | Tobacco  Fossil fuels | Reference chained (from Petticrew et al., 2017) | Overview | ‘view point’ | No |
| Egilman and Billings, 2005 Abuse of epidemiology: automobile manufacturers manufacture a defense to asbestos liability | Chemicals and manufacturing | Reference chained (from Egilman and Bohme 2005) | Primary study | No categorisation | No |
| Egilman & Bohme, 2005. Over a barrel: corporate corruption of science and its effects on workers and the environment | Chemicals and manufacturing  Extractive | Reference chained (from Bero, 2013) | Overview | No categorisation (introduction to special issue) | No |
| Hanauer et al., 1995 Lawyer control of internal scientific research to protect against products liability lawsuits | Tobacco | Reference chained (from White and Bero, 2010) | Primary study | No categorisation (documents analysis) | No |
| Jernigan, 2012 Global alcohol producers, science and policy: the case of the International Center for Alcohol Policies | Alcohol | Reference chained (from Savell, 2015) | Primary study | No categorisation | No |
| McGarity and Wagner 2008 Bending Science, How Special Interests Corrupt Public Health Research | Tobacco  Pharmaceuticals and medical technologies  Chemicals/manufacturing  Fossil fuels  Food and drink | Reference chained (from Pinto, 2017) | Overview | Book | No |
| Michaels and Monforton, 2005 Manufacturing Uncertainty: Contested Science and the Protection of the Public’s Health and Environment | Tobacco  Chemicals and manufacturing  Pharmaceuticals and medical technologies | Ref chained (from UCS, 2007) | Overview | No categorisation | No |
| Nestle, 2013 Food Politics: How the Food Industry Influences Nutrition and Health | Food and drink | Reference chained (from Kearns et al 2015) | Overview | Book | No |
| Ong and Glantz, 2001. Constructing “sound science” and “good epidemiology”: tobacco, lawyers and public relations firms | Tobacco | Reference chained (from Bero 2013) | Overview | No categorisation | No |
| Rothman et al., 2009 Professional medical associations and their relationships with industry – a proposal for controlling conflict of interest | Pharmaceuticals and medical technologies | Reference chained (from Akl and Khamis, 2019) | Overview | ‘Special Communication’ | No |
| Sass 2005 Industry Efforts to weaken the EPA’s classification of the carcinogenicity of 1-3 butadiene | Chemicals and manufacturing | Reference chained (from Egilman 2005) | Primary study | No categorisation | No |
| Smith et al. 2010b. “Working the System”: British American Tobacco’s Influence on the European Union Treaty and Its Implications for Policy: An Analysis of Internal Tobacco Industry Documents | Tobacco  Chemicals and manufacturing | Reference chained (from Bero 2013) | Primary study | No categorisation (documents analysis) | No |
| Smoke free Partnership, 2010. The origin of EU Better Regulation – The Disturbing Truth | Tobacco  Chemicals and manufacturing | Reference chained (from Smith et al., 2015) | Overview | Report | No |
| Union of Concerned Scientists, 2007. Smoke, Mirrors and Hot Air. How Exxon Mobil uses Big Tobacco’s tactics to manufacture uncertainty on climate science | Fossil fuels  Tobacco | Reference chained (from Bero, 2013) | Primary study | Report | No |
| Studies identified through expert recommendation | | | | | |
| Babor 2009 Alcohol research and the alcoholic beverage industry: issues, concerns and conflicts of interest | Alcohol | Expert recommendation | Overview | ‘review’ (documents analysis) | No |
| Bero, 2013. Tobacco industry manipulation of research | Tobacco  Chemicals and manufacturing | Expert recommendation | Overview | Report | No |
| Bialous and Yach (2001) Whose standard is it anyway? How the tobacco industry determines the International Organization for Standardization (ISO) standards for tobacco and tobacco products | Tobacco | Expert recommendation | Primary study | No categorisation (documents analysis) | No |
| Bialous and Glantz (2002) ASHRAE Standard 62: tobacco industry’s influence over national ventilation standards | Tobacco | Expert recommendation | Primary study | No categorisation (documents analysis) | No |
| Bjornberg et al, 2017 Climate and environmental science denial: a review of the scientific literature published in 1990-2015 | Fossil fuels | Expert recommendation | Overview | ‘systematic review’ | No |
| Brandt, 2012. Inventing conflicts of interest: a history of tobacco industry tactics | Tobacco | Expert recommendation | Overview | No categorisation | No |
| Cassidy et al., 2013 Fair Game: Producing gambling research | Gambling | Expert recommendation | Primary study | Report | Yes (the authors received research funds from an organisation that receives funds from the gambling industry) |
| Dunlap and McCright, 2010. Climate change denial: sources, actors and strategies | Fossil fuels | Expert recommendation | Overview | Book chapter | No |
| Dunlap and McCright, 2011. Organised climate change denial | Fossil fuels | Expert recommendation | Overview | Book chapter | No |
| Granheim et al., 2017 Interference in public health policy: examples of how the baby food industry uses tobacco industry tactics | Tobacco  Food and drink | Expert recommendation | Overview | ‘literature review’ | No |
| Gruning et al., 2006. Tobacco industry influence on science and scientists in Germany | Tobacco | Expert recommendation | Primary study | No categorisation (documents analysis) | No |
| Kearns, et al. 2015. Sugar industry influence on the scientific agenda of the National Institute of Dental research’s 1971 National Caries Program: A Historical Analysis of Internal Documents | Food and drink | Expert recommendation | Primary study | ‘a historical analysis of internal documents’ | No |
| Kearns et al., 2016 Sugar industry and coronary heart disease research – a historical analysis of internal industry documents | Food and drink | Expert recommendation | Primary study | ‘a historical analysis of internal industry documents’ | No |
| Kirsch, 2014. Corporate Science. Chapter 4 of Mining Capitalism – the relationship between corporations and their critics | Tobacco  Pharmaceuticals and medical technologies  Fossil fuels  Extractive | Expert recommendation | Overview | Book chapter | No |
| Lee et al., 2012 The vector of the tobacco epidemic: tobacco industry practices in low and middle-income countries | Tobacco | Expert recommendation | Overview | ‘systematic review’ | No |
| PANUK et al., 2020 Toxic Trade – how trade deals threaten to weaken UK pesticide standards | Chemicals and manufacturing | Expert recommendation | Overview | Report | No |
| McDaniel et al., 2006 Philip Morris’s Project Sunrise: weakening tobacco control by working with it | Tobacco | Expert recommendation | Primary study | No categorisation (documents analysis) | No |
| Michaels, 2008 Manufactured Uncertainty: Contested Science and the Protection of the Public’s Health and Environment | Tobacco  Pharmaceuticals and medical technologies  Chemicals and manufacturing  Fossil fuels | Expert recommendation | Overview | Book chapter | No |
| Nestle, 2015. Soda Politics | Food and drink | Expert recommendation | Overview | Book | No |
| Petticrew et al., 2017. How alcohol industry organisations mislead the public about alcohol and cancer | Alcohol | Expert recommendation | Primary study | Qualitative analysis of websites and documents (documents analysis) | No |
| Peeters et al., 2015 The revision of the 2014 European tobacco products directive: an analysis of the tobacco industry’s attempts to ‘break the health silo’ | Tobacco | Expert recommendation | Primary study | Document analysis and stakeholder interviews | No |
| Smith et al., 2015. Lowest common denominator. How the proposed EU-US trade deal threatens to lower standards of protection from toxic pesticides | Chemicals and manufacturing | Expert recommendation | Overview | Report | No |
| Smith et al., 2015. Corporate coalitions and policymaking in the European Union: how and why British American Tobacco promoted ‘Better Regulation’ | Tobacco  Chemicals and manufacturing | Expert recommendation | Primary study | Article ‘drawing on documentary and interview data’ | No |
| Thomas et al, 2018 A public health framework for assessing gambling industry strategies and tactics | Gambling | Expert recommendation | Primary study | Report | Yes (authors declare they have received research funds from an organisation which is funded via hypothecated taxes from the gambling industry) |
| Ulucanlar et al., 2014 Representation and Misrepresentation of Scientific evidence in contemporary tobacco regulation: a review of tobacco industry submissions to the UK government consultation on standardised packaging | Tobacco | Expert recommendation | Primary study | No categorisation (documents analysis) | No |
| Union of Concerned Scientists, 2012. Heads they win, tails you lose: how corporations corrupt science at the public’s expense | Tobacco  Extractive  Pharmaceuticals and medical technologies  Chemicals and manufacturing  Fossil fuels  Food and drink | Expert recommendation | Overview | Report | No |
| White & Bero, 2010. Corporate manipulation of research: strategies are similar across five industries | Tobacco  Extractive  Chemical and manufacturing  Pharmaceuticals and medical technologies | Expert recommendation | Primary study | No categorisation (documents analysis) | No |
| Studies identified from web alerts | | | | | |
| Akl and Khamis, 2019 The intersections of industry with the health research enterprise | Pharmaceuticals and medical technologies  Food and drink | Web alert | Overview (of industry activities) | Commentary | No |
| Connor and Kypri, 2018, The alcohol industry, the government and the alleged defamation of public health advocates: A New Zealand case study | Alcohol | Web alert | Commentary | Commentary | No |
| Cullen et al., 2017. ‘Half-cut’ science: a qualitative examination of alcohol industry actors’ use of peer-reviewed evidence in policy submissions on Minimum Unit Pricing | Alcohol | Web alert | Primary study | No categorisation (documents analysis) | No |
| Fabbri et al., 2018 The influence of industry sponsorship on the research agenda: a review | Tobacco  Pharmaceuticals and medical technologies  Food and drink  Chemical and manufacturing  Alcohol  Extractive | Web alert | Overview | ‘scoping review’ | No |
| Livingstone, 2018 A case for clean conferences in gambling research | Gambling | Web alert | Commentary | Commentary | Yes (the author declares they have received research funds from organisations funded via hypothecated taxes from the gambling industry) |
| Petticrew et al., 2017. ‘Nothing can be done until everything is done’: the use of complexity arguments by food, beverage, alcohol and gambling industries | Food and drink  Alcohol  Gambling | Web alert | Primary study | Primary study | No |
