# Supplementary material for: The Science for Profit Model—How and why corporations influence science and the use of science in policy and practice
Source: PLoS One. 2021 Jun 23;16(6):e0253272. doi: 10.1371/journal.pone.0253272 (PMC8221522; doi:10.1371/journal.pone.0253272)
Supplement: S2 Appendix — (DOCX) [file pone.0253272.s002.docx]

**S2 Appendix. Sectors of industry investigated.**

| Industry sector | Examples of sub-divisions within sectors (where authors have specified) | Number of included studies that investigated this sector |
| --- | --- | --- |
| Tobacco | Multi-national tobacco producers | 34 |
| Pharmaceuticals and medical technologies | - Pharmaceutical manufacturers - Medical device manufacturers | 21 |
| Food and drink | - Multi-national developers, manufacturers and producers including those producing: - Confectionary - Sugar-sweetened beverages - Breakfast cereals - Eggs - Meat - Infant formula - Salt - Genetically modified food - Food additives (e.g. cane and beet sugar, artificial sweeteners, fat substitutes) - Dietary supplements | 16 |
| Chemicals and manufacturing | - Manufacturers that produce harmful or potentially harmful substances which have been used in consumer products (e.g. lead – used in leaded petrol and paint; vinyl chloride - a substance used in household products such as hair sprays, shower curtains and car upholstery; and formaldehyde – a substance used in household products such as mattresses, air fresheners and furniture) - Manufacturers that produce harmful or potentially harmful substances for use in agriculture e.g. agrochemical companies producing pesticides such as those which contain carcinogenic and endocrine-disrupting chemicals - Manufacturers who have used harmful or potentially harmful substances as part of their manufacturing processes (e.g. butadiene – which has been used in the manufacture of rubber and paint; asbestos – which has been used in brake linings in automotive brake manufacturers; and nylon particles in textiles factories causing ‘flock lung’ in workers) - Manufacturers who produce harmful or potentially harmful substances as by-products of manufacturing processes (e.g. fluoride, since its inhalation may cause harm to workers) | 17 |
| Alcohol | - Multi-national alcohol producers | 11 |
| Fossil fuels | - Extractors and distributors of fossil fuels (oil, coal and natural gas) | 12 |
| Extractive | - Corporations conducting non-fossil-fuel extraction (e.g. copper, gold, zinc and asbestos mining) | 5 |
| Gambling | - Gambling operators | 4 |

Nb. other industries attempting to influence science and/or the use of science were mentioned briefly in the literature but have been omitted from our current analysis due to a paucity of data. These included the banking industry (Pinto, 2017), animal biotechnology (Fabbri et al., 2018) and tanning industries (Michaels and Monforton, 2005). Bero (2013) also outlines that the tobacco industry worked to mobilise allied industries including fishing and waterworks industries in its attempts to embed data access and data quality legislation in the US, but it is unclear to what extent these industries became involved. Smith et al. (2015) outline industries that were recruited by British American Tobacco and the European Policy Centre to illustrate support for their efforts to embed structured risk assessment in EU policymaking and these include corporations from the construction industry, multinational food and other retail, telecommunications, aerospace and arms, nuclear power and media corporations.
