# Supplementary material for: The Science for Profit Model—How and why corporations influence science and the use of science in policy and practice
Source: PLoS One. 2021 Jun 23;16(6):e0253272. doi: 10.1371/journal.pone.0253272 (PMC8221522; doi:10.1371/journal.pone.0253272)
Supplement: S3 Appendix — (DOCX) [file pone.0253272.s003.docx]

## S3 Appendix. Macro, meso and micro strategies used by industry to influence science and the use of science in policy and practice – purposes, supporting evidence, use by different industries, features and examples.

| Macro and meso strategies (purpose, supporting evidence, and use by each industry) | Micro strategies | Features and examples |
| --- | --- | --- |
| Macro Strategy A. Influence the conduct^[[1]](#footnote-1)^ and publication of science to skew evidence bases in industry’s favour | | |
| 1. Fund and undertake “safe” research   \| Purpose: to ensure that research funded by industry maximises benefits and minimises harm to industry \| \| --- \|  \| Supporting evidence: (1-22) \| \| --- \|  \| Tobacco \| Pharma \| \| --- \| --- \| \| Alcohol \| Fossil fuels \| \| Food and drink \| Extractive \| \| Chemicals/manufact. \| Gambling \| | - 1.1. Fund and undertake “safe” research which distracts attention from industry harms, frames industry and industry products as part of the “solution”, and promotes interventions that minimise damage to product sales - 1.2. Commission lawyers and public relations (PR) firms to manage research programmes to ensure research is “safe”^[[2]](#footnote-2)^ - 1.3 Fund and undertake research to identify or demonstrate public perceptions | - 1.1 Fund and undertake “safe” research, which may be methodologically sound but acts to skew the evidence base by distracting attention away from industry harms, framing industry and industry products as part of the “solution” and/or promoting interventions that minimise damage to product sales by e.g.:   - 1.1.1 Suggesting causes of harm other than that of the corporate product or practice: e.g. tobacco industry grants focusing on non-tobacco causes of cancer such as infection, nutrition, hormones (Pinto, 2017, p.57); e.g. Coca-Cola funding obesity research focused on physical activity, downplaying the role of its products in obesity (Fabbri et al., 2018, p.4) - 1.1.2 Suggesting problems of corporate harm are problems “of the individual” e.g. research focusing on “problem” gambling and the “problem gambler” (Cassidy et al., 2013, p.29)   1.1.3 Focusing on reducing harm from, rather than intake/use of products/practices e.g. research on vaccines to prevent tooth decay (rather than research on sugar) (Kearns et al., 2015, p.12)   - 1.1.4 Focusing on supposed *benefits* of industry products or practices over possible harms: e.g. the alcohol industry-funded International Center for Alcohol Policies focusing on research concerning the pleasurable effects of alcohol (Jernigan 2012, p.82), and the tobacco industry focusing on the psychosocial benefits of smoking (Gruning et al., 2006, p.23) - 1.1.5 Focusing on industry products as solutions to public health problems (rather than broader public health interventions) e.g. food industry focusing on specific nutrients rather than whole foods, and pharmaceutical industry focusing on drugs and devices rather than behavioural interventions (Fabbri et al., 2018, p.8) - 1.1.6 Suggesting regulation of industry products or practices is undesirable and/or promoting the supposed benefits of policy and practice interventions that do not involve regulation of industry e.g. the alcohol industry favouring research with a focus on solutions such as alcohol education programmes (for which the industry knew there was limited evidence of effectiveness) rather than evidence-based alcohol control measures which would affect industry’s profitability (such as taxation and limits on availability) (Babor, p.40; Babor and Robaina, p.208) - 1.1.7 Promoting industry as part of the solution e.g. the alcohol industry funding research arguing that industry’s involvement in alcohol education programmes (which the author themselves concluded have not been shown to be effective) is evidence that ‘industry sponsored bodies are actively participating in preventing the misuse of their products’ (Babor, 2009, p.40) and that school-based education should be conducted in partnership with industry (Babor and Robaina, 2013, p.208)   - 1.2 Commission lawyers and PR firms to manage research programmes, and to advise on which grant proposals to fund in order to ensure that research is “safe” and maximally beneficial to industry: e.g. the tobacco industry used law firm Covington and Burling to manage their second-hand smoke research programme and commission research suggesting poor indoor air quality was caused by factors other than smoking, and that improved ventilation would eliminate health harms linked to passive smoking (Lee et al., 2012, p.124); e.g. PR firm Hill and Knowlton designing a research programme for a chemical manufacturer to create doubt about the harms caused by industrial by-product, dioxin (McGarity & Wagner, 2008, p.215)  -1.3 Fund and undertake research to identify or demonstrate public perceptions:   - 1.3.1 Survey the public on matters concerning corporate products or practices, in order to inform industry responses e.g. The Sugar Research Foundation’s plan to conduct opinion polls “to learn what public concepts we should reinforce and what ones we need to combat through our research” (Kearns et al., 2016, p.E2) - 1.3.2 Survey the public on scientific issues to demonstrate public support for industry positions e.g. the tobacco industry sponsoring an opinion poll to demonstrate support for industry perspectives concerning evidence standards in epidemiology (Bero, 2013, p.163) |
| 2 Covertly undertake or prevent “risky” industry research   \| Purpose: To ensure that potentially unfavourable research can be withheld from the evidence base, or to prevent the existence of such research altogether^[[3]](#footnote-3)^ \| \| --- \|  \| Supporting evidence: (4, 10, 16, 23, 24) \| \| --- \|  \| Tobacco \| Pharma \| \| --- \| --- \| \| Alcohol \| Fossil fuels \| \| Food and drink \| Extractive \| \| Chemicals/manufact. \| Gambling \| | - 2.1 Covertly undertake “risky” research so that it can be hidden or abandoned - 2.2 Prevent “risky” industry research from being undertaken | -2.1 Covertly undertake “risky” research so that it can be hidden or abandoned:   - 2.1.1 Conducting research internally so that it can be hidden: e.g. the tobacco industry conducting research (into chemical additives in cigarettes) internally in order to be able to keep it secret if it were unfavourable (some of this research did show increased emissions of toxins) (Bero, 2013, p.161) - 2.1.2 Failing to adequately register clinical trials: e.g. the pharma industry failing to register clinical trials (Schott et al., 2010a) - 2.1.3 Abandoning research projects which produce unfavourable findings e.g. at Philip Morris’ secret lab looking at the addictiveness of nicotine in rats, findings were unfavourable and so the lead scientist was ordered to “close down his laboratory, to kill the animals, to suspend all further investigation of possibly less toxic or harmful alternatives to nicotine, never try to publish or discuss his work on addicting rats, and to find work elsewhere” (McGarity and Wagner, 2008, p.111).   - 2.2 Prevent “risky” industry research from being undertaken e.g. tobacco industry scientists at Brown & Williamson (B&W) advising British American Tobacco (BAT) scientists not to conduct research on certain topics such as inhalation (presumably because they feared the research would lead to unfavourable results) (Hanauer, 1995, p.238) |
| 3. Control design & analysis of industry-funded science to ensure favourable results   \| Purpose: To maximise the existence of favourable research and minimise the existence of unfavourable research in the evidence base \| \| --- \|  \| Supporting evidence: (1, 4, 10-12, 14, 16, 19, 22, 24-31) \| \| --- \|  \| Tobacco \| Pharma \| \| --- \| --- \| \| Alcohol \| Fossil fuels \| \| Food and drink \| Extractive \| \| Chemicals/manufact. \| Gambling \| | - 3.1 Control the design and analysis of industry-funded primary studies - 3.2 Control the design and analysis of industry-funded evidence syntheses | - 3.1 Control the design and analysis of primary studies:   - 3.1.1 Controlling design to ensure favourable results: e.g. the pharmaceutical industry’s use of inappropriate comparators such as placebos rather than active controls (Al-Badriyeh et al., 2017, p.7); a formaldehyde industry-funded study which found no association between the chemical and cancer in rats (but exposed far fewer rats, for a much shorter time period when compared with the study it was attempting to counter) (Union of Concerned Scientists, 2012, p.150); e.g. the extractive industry failing to collect sufficient background data so that environmental harms cannot be properly measured (Kirsch, 2014, p.145); e.g. tobacco industry-funded research containing data which had been “altered and fabricated” and therefore concluded that “moderate” amounts of indoor smoking were safe (Bero, 2013, p.157) - 3.1.2 Controlling analysis to ensure favourable results: e.g. inappropriate use of statistics in extractive industry research, specifically, calculating mean levels of toxicity, when what is important is the frequency with which safety levels are exceeded (Kirsch, 2014, p.140)   - 3.2 Control the design and analysis of evidence syntheses:   - 3.2.1 Controlling design of evidence syntheses: e.g. controlling eligibility criteria in order to influence which papers are included in a synthesis (cherry-picking papers) such as the American Plastics Council review on bisphenol A (BPA – a plastic used in e.g. food cans) which found no evidence of toxicity at low levels. Critics said the review only looked at a minority of the available studies (Michaels, 2008); e.g. in industry-funded reviews of the association between exposure to asbestos in car brakes and cancer, the authors selectively chose the data within the included studies which demonstrated average (acceptable) exposure, while data showing greater exposure were ignored (Egilman and Billings, 2005, p.362) - 3.2.2 Controlling analysis of evidence syntheses: e.g. an automobile industry funded meta-analyses on asbestos and cancer which re-analysed data from included studies, combining studies which had lung cancer control groups and studies which had population control groups, taking the mean odds ratio, which had the effect of making the link between exposure to asbestos and cancer appear smaller (Egilman and Billings, 2005, p.365) |
| 4. Shape and undermine external research   \| Purpose: To ensure any research conducted by external organisations aligns with industry interests \| \| --- \|  \| Supporting evidence: (1, 7, 8, 12, 13, 15, 21, 32-35) \| \| --- \|  \| Tobacco \| Pharma \| \| --- \| --- \| \| Alcohol \| Fossil fuels \| \| Food and drink \| Extractive \| \| Chemicals/manufact. \| Gambling \| | - 4.1 Shape external (e.g. governmental) organisations’ research priorities through access, funding, and political power^[[4]](#footnote-4)^ - 4.2 Attempt to block the funding of potentially unfavourable independent research - 4.3 Deliberately obstruct independent data collection | - 4.1 Shape external (e.g. governmental) organisations’ research priorities through access, funding, and political power:   - 4.1.1 Gaining access to public research bodies e.g. The Sugar Research Foundation altering the research priorities of the National Institute of Dental Research through influencing its expert panel (Kearns et al., 2015, p.10) - 4.1.2 Funding grant-making bodies which were set up independently from industry in order to shape their research priorities e.g. the gambling industry’s voluntary contributions to intermediary the Responsible Gambling Trust appear to have shaped the research priorities (namely on to “problem gambling”) of the grant-making body (Cassidy et al., 2013, p.66) - 4.1.3 Using political power to shape state research agendas e.g. Philip Morris lobbying to divert state tobacco control funding to non-tobacco research (McDaniel et al, 2006, p.218)   - 4.2 Attempt to block the funding of potentially unfavourable external research: e.g. Philip Morris using political power to attempt to prevent funding of WHO’s International Agency for Research on Cancer (IARC) (Lee et al., 2012); e.g. the Sugar Association asking Congress not to fund World Health Organization (Brownell and Warner, 2009).  - 4.3 Deliberately obstructing independent data collection: e.g. the gambling industry attempting to discourage researchers from collecting data by refusing to circulate their data collection tools (Cassidy et al., 2013, p.72) and avoiding meeting with researchers (e.g. ignoring requests for interviews or continually postponing them) (ibid, p.76) |
| 5. Ensure favourable research is heavily represented in the evidence base   \| Purpose: To maximise the extent to which the science on industry products and practices favours industry arguments \| \| --- \|  \| Supporting evidence: (2-4, 8, 11-13, 16, 19, 22, 27, 28, 30, 31, 35-42) \| \| --- \|  \| Tobacco \| Pharma \| \| --- \| --- \| \| Alcohol \| Fossil fuels \| \| Food and drink \| Extractive \| \| Chemicals/manufact. \| Gambling \| | - 5.1 Maximise the presence of industry-funded publications in the peer-reviewed literature - 5.2 Fund or create journals to have influence over what is published - 5.3 Create publications which emulate peer-reviewed/quality science | - 5.1 Maximise the presence of industry-funded publications in the peer-reviewed literature by:   - 5.1.1 Identifying peer-reviewed publications that accept industry-funded science and publishing favourable science^[[5]](#footnote-5)^ there: e.g. the alcohol industry-funded Australian Wine Research Institute publishing industry-favourable papers on the relationship between alcohol and health in peer-reviewed addiction science journals (Babor, 2009, p.41) - 5.1.2 Repeatedly publishing the same research: e.g. one trial on the efficacy of antipsychotic drug, Risperdal, was published in six different journals using different author names (Union of Concerned Scientists, 2012, p.17). - 5.1.3 Using “ghost-writing” companies to produce large numbers of papers (ostensibly written by experts in the field) e.g. an independent review of evidence on antidepressant Zoloft showed 55/96 papers in the evidence base were co-ordinated by one ghost-writing company, and the “authors” of these papers “had published on average three times more articles than the other authors” (McGarity & Wagner, 2008, p.79)   - 5.2 Fund or create journals to have influence over what is published:   - 5.2.1 Funding existing journals: e.g. the Air Hygiene Foundation (a silica industry-funded body) funded the Journal of Industrial Hygiene and Toxicology, and “in return, the Journal…assisted the managing Director of the Foundation in the publication of his own abstracts” (White and Bero, 2010, p.117) - 5.2.2 Creating and populating own industry journals such as the tobacco industry-funded “Tobacco and Health Research” journal which was distributed to health professionals (White and Bero, 2010, p.126)   - 5.3 Create publications which emulate peer-reviewed/quality science such as:   - 5.3.1 Symposia proceedings: funding and holding symposia in order to enable the publication of (and subsequent citation of) proceedings which mimic peer-reviewed literature but do not have to go through peer-review e.g. the pharmaceutical industry publishing symposia proceedings which favour certain drugs (Bero, 2013, p.159); the tobacco industry publishing symposia proceedings concerning second-hand smoke (Pinto, 2017, p.59) - 5.3.2 Reports: e.g. the Heartland Institute (funded by the fossil fuels industry) creating publications designed as alternatives to assessments published by the Intergovernmental Panel on Climate Change (Dunlap and McCright, 2010, p.254) - 5.3.3 Abstracts of unpublished research: e.g. the industry-funded Alcohol Beverage Medical Research Foundation acting as an abstracting service - publishing lists of abstracts of current research but selecting these on the basis of their mission, which was to emphasise the benefits of moderate drinking (Babor, 2009, p41) |
| 6. Control reporting and suppress publication of unfavourable science   \| Purpose: To prevent potentially unfavourable science (both industry-funded and independent) from becoming part of the evidence base \| \| --- \|  \| Supporting evidence: (1, 3, 4, 7, 10, 12, 14, 16, 17, 19, 22-27, 29-32, 35, 36, 42, 43) \| \| --- \|  \| Tobacco \| Pharma \| \| --- \| --- \| \| Alcohol \| Fossil fuels \| \| Food and drink \| Extractive \| \| Chemicals/manufact. \| Gambling \| | - 6.1 Control the way in which unfavourable industry science is reported within publications - 6.2 Suppress publication of unfavourable science | - 6.1 Control the way in which science is reported within publications through:   - 6.1.1 Selective reporting: e.g. Pfizer selectively reporting outcomes from studies on epilepsy drug, Neurontin, and failing to report the increased risk of suicide (Union of Concerned Scientists, 2012, p.16) - 6.1.2 Obtaining legal advice on reporting: employing lawyers to suggest edits to documents to prevent anything unfavourable from being published e.g. tobacco industry lawyers suggesting altering documents to reinforce the idea of a “controversy” around the dangers of smoking (Hanauer et al., 1995, p.236); e.g. lawyers for the asbestos industry suggesting changes to a publication which showed high rates of asbestosis in workers, recommending that "all of the favourable aspects of the survey be included” (McGarity and Wagner, 2008, p.76) - 6.1.3 Pressuring industry scientists in order to influence write-up: e.g. the gambling industry disagreeing with researchers’ conclusions and demanding the removal of unfavourable sections (Cassidy et al., 2013, p.79); pressuring authors to alter their reporting in publications already published e.g. authors of an industry-funded study on vinyl chloride published it in the American Journal of Industrial Medicine before the industry had been consulted. After communications with the Chemical Manufacturers Association the authors wrote to the journal and retracted some of the findings, saying that “our finding of an excess of brain cancer among US vinyl chloride workers reported earlier was not likely related to the chemical” (White and Bero, 2010, p. 117).   - 6.2 Suppress publication of unfavourable science through:   - 6.2.1 Not publishing trials/unfavourable results: e.g. Glaxo Smith Kline funding five clinical trials on its antidepressant, Paxil, but only publishing one (the other four showed unfavourable results including increased risk of suicide) (Union of Concerned Scientists, 2012, p.13) - 6.2.2 Securing legal rights to publication e.g. the gambling industry requiring scientists to sign non-disclosure agreements which restrict publication (Cassidy et al., 2013, p.79*)* - 6.2.3 Pressuring scientists in order to prevent write-up: demanding researchers withdraw papers e.g. Philip Morris demanding a researcher withdraw a paper which illustrated the addictiveness of nicotine in mice (McGarity and Wagner, 2008, p.111); threats of legal action against researchers e.g. scientist Betty Dong was told not to publish Boots-funded research about drug Synthroid and both she and her university were threatened with legal action (UCS, 2012, p.14)^[[6]](#footnote-6)^ - 6.2.4 Pressure on journals: using financial pressure to prevent publication of unfavourable independent science e.g. the journal “Alcohol and Alcoholism” whose industry sponsor pressured them to reduce the number of papers on the harmful effects of alcohol (Babor, 2009, p.41); sending negative reviews to journals e.g. manufacturers of asthma drug Fenoterol contacted The Lancet with multiple negative reviews of a study they wished to suppress (McGarity & Wagner, 2008, p.141); threatening legal action against journals e.g. the manufacturer of the AneuRx stent (a medical device) threatened to sue the Journal of Vascular Surgery, claiming that the data supplied to the FDA on which the independent unfavourable science was based were proprietary trade secrets (McGarity & Wagner, 2008, p.115). - 6.2.5 Using political power to prevent publication of independent research: e.g. The Sugar Association attempting to block a report written by the WHO outlining a global strategy on diet and physical activity. The Sugar Association “criticized both the science and the process by which the report was prepared and asked that it be stopped, or at the very least, delayed” (Brownell and Warner 2009 p.275) |
| Macro Strategy B. Influence the interpretation of science to undermine unfavourable science and create a distorted picture of the evidence base | | |
| 7. Develop and promote criteria and concepts for critiquing science which can be used to further industry arguments   \| Purpose: To establish criteria and concepts for determining “quality” science and scientific “proof” that can be used to undermine unfavourable public health science in order to prevent legislation \| \| --- \|  \| Supporting evidence: (4, 19, 22, 26, 31, 35, 37, 44-48) \| \| --- \|  \| Tobacco \| Pharma \| \| --- \| --- \| \| Alcohol \| Fossil fuels \| \| Food and drink \| Extractive \|  \| Chemicals/manufact. \| Gambling \| \| --- \| --- \| | - 7.1 Develop criteria for the conduct and interpretation of science (including determining scientific “proof”) with the intention that these can be used to undermine unfavourable science - 7.2 Adopt the concepts of “junk science” and “sound science” for use in undermining unfavourable science and promoting industry science - 7.3 Fund and coordinate public relations campaigns to promote these industry-friendly criteria and concepts to key stakeholders^[[7]](#footnote-7)^ | - 7.1 Develop criteria for the conduct and interpretation of science (including determining scientific “proof”) with the intention that they can then be used to undermine unfavourable science: e.g. the Chemical Manufacturers Association’s “Guidelines for Good Epidemiology Practices” were ostensibly created to improve the quality of the conduct and evaluation of epidemiological studies, but were a pre-cursor to a campaign intending to undermine unfavourable epidemiological studies as a whole (Ong and Glantz, 2001, p.1750); e.g. guidelines drawn up by Shook, Hardy and Bacon, lawyers of Philip Morris, which went further, attempting to determine the criteria for establishing scientific proof, arguing that “odds ratios of 2 or less are highly questionable” (other drafts of the document said they were likely to be “artefactual” i.e. an error) and that “a statistical significant association is not strong enough evidence for causation to warrant regulatory action” (Ong and Glantz, 2001, p.1751). - 7.2 Adopt the concepts of “junk science” and “sound science”: co-opting the term “junk science” as a loosely-defined phrase which could be used to attack any science deemed unfavourable to industry e.g. in the industry-funded Manhattan Institute’s book “Galileo’s Revenge” where such science is described as “the mirror image of real science, with much of the same form but none of the substance…it is a hodgepodge of biased data, spurious inference…data dredging, wishful thinking, truculent dogmatism, and now and again, outright fraud” (Bingham et al, 2003, p.6); co-opting the term “sound science” as the opposite of “junk science” e.g. through The Advancement of Sound Science Coalition (see below) (Ong and Glantz, 2001). - 7.3 Fund and coordinate public relations campaigns to promote the use of these industry-friendly criteria and concepts to key stakeholders: e.g. in the early 1990s, the tobacco industry launching public relations campaigns in the US and the EU controlled by lawyers and PR firms to promote the rhetoric of “sound science”, “junk science” and “good epidemiology” to consumers of epidemiology (scientists, policymakers, regulators), including through the creation of front group The Advancement for Sound Science Coalition (TASSC). The intended outcome was to shape perceptions of the standards of evidence needed to establish causation of harm, discredit existing and future research on industry harms, and ultimately to prevent unfavourable legislation (Ong and Glantz, 2001) |
| 8. Obtain and reanalyse raw data from unfavourable science   \| Purpose: To undermine the findings from unfavourable studies \| \| --- \|  \| Supporting evidence: (4, 12, 16, 26, 27, 35, 36, 45, 49) \| \| --- \|  \| Tobacco \| Pharma \| \| --- \| --- \| \| Alcohol \| Fossil fuels \| \| Food and drink \| Extractive \| \| Chemicals/manufact. \| Gambling \| | - 8.1 Obtain raw data underlying unfavourable research by enabling data access litigation, and pressuring or litigating against scientists - 8.2 Re-analyse independent data including that acquired in these ways to refute unfavourable findings | - 8.1 Work to obtain the raw data underlying unfavourable research in order to re-analyse them^[[8]](#footnote-8)^ by:   - 8.1.1 Enabling data access legislation e.g. Philip Morris initiating a campaign which led to the implementation of the Data Access Act, which meant that publicly funded research had to make its raw data public (Baba et al., 2005) - 8.1.2 Pressuring researchers: e.g. the pharmaceutical industry contacting a university dean to demand access to raw data of a pre-publication study. The study suggested new antihypertensive drugs were associated with heart attacks, and the researcher had just presented the findings at a conference (McGarity and Wagner, 2008, p.142) - 8.1.3 Litigating against researchers e.g. tobacco corporation RJ Reynolds litigating in attempts to gain access to data (including lab books and personal data of participants) from a study suggesting children had positive reactions to industry mascot, ‘Joe Camel’ (McGarity and Wagner, 2008, p.172)   - 8.2 Re-analyse datasets from unfavourable research in order to refute unfavourable findings: e.g. the chromium industry obtaining and re-analysing the EPA’s raw data from a study showing the harm caused to workers from chromium in order to challenge its validity (McGarity and Wagner, 2008, p.136); e.g. a product defence firm conducting a re-analysis of data from a Center for Disease Control study (which showed a link between exposure to beryllium – a metal used in weapons manufacture – and lung cancer), changing some parameters so that the “elevation of lung cancer rates was no longer statistically significant” (Michaels, 2008). |
| 9. Attack and misrepresent science^[[9]](#footnote-9)^     \| Purpose: To undermine and distort understandings of unfavourable science \| \| --- \|  \| Supporting evidence: (2-4, 6, 7, 9, 11, 12, 14-19, 21, 22, 24-27, 31-33, 35, 37, 38, 40, 41, 44-48, 50-52) \| \| --- \|  \| Tobacco \| Pharma \| \| --- \| --- \| \| Alcohol \| Fossil fuels \| \| Food and drink \| Extractive \| \| Chemicals/manufact. \| Gambling \| | - 9.1 Attack the methods of unfavourable science (tailoring the criteria depending on the nature of the science to be attacked) - 9.2 Label unfavourable science “junk” - 9.3 Misrepresent single pieces of evidence - 9.4 Misrepresent whole evidence bases - 9.5 Mispresent expert consensus | - 9.1 Attack the methods of unfavourable science^[[10]](#footnote-10)^ (using criteria that can be tailored depending on the nature of the science to be attacked) by:   - 9.1.1 Attacking studies based on issues of data collection, analysis and reporting (insisting on methodological perfection) e.g. the tobacco industry criticising a US Environmental Protection Agency risk assessment (which concluded that second-hand smoke was carcinogenic) for the way it measured exposure and controlled for bias and confounding (Bero, 2013, p.153) - 9.1.2 Attacking whole classes or types of evidence e.g. the sugar industry discounting studies (epidemiological/animal/mechanistic etc.) based on the class of evidence because their findings are unfavourable to industry, rather than because of issues of rigor (Kearns et al., 2016, p.E3); utilising double standards around critiquing evidence: e.g. the tobacco industry criticising qualitative research on attitudes/experience etc. but then praising other studies with these methods since their results were favourable to industry (Ulucanlar et al., 2014, p.7) - 9.1.3 Insisting on classes or types of evidence that do not or cannot exist: e.g. chemicals and manufacturing industry insisting on data which is very difficult to produce, such as that within which individuals are exposed to the substance in question only (Egilman and Billings, 2005, p.361); Insisting the methods of existing evidence (whatever its type) mean that causation cannot be established “for example, when a suspect carcinogen is found to cause cancer in human epidemiologic studies but not in animals, companies argue that animal studies are required to prove causation. On the other hand, when animal studies are positive and human epidemiology is incomplete or negative, companies argue that human evidence is required before the government can regulate the substance and before workers and others can be compensated” (Egilman and Billings, 2005, p.361).   - 9.2 Label unfavourable science “junk” e.g. in the passive smoking debate, the tobacco industry labelling the Environmental Protection Agency’s report on passive smoking as “junk science”, and then going to use this moniker for any (often legitimate) science which was potentially unfavourable to industry (Pinto, 2017, p.59)  - 9.3 Misrepresent single pieces of evidence by misquoting, selectively quoting, or misinterpreting studies: e.g. the tobacco industry misleadingly quoting studies on standardised packaging of cigarettes including by using the “tweezer method” to pick out favourable pieces of text to quote (Ulucanlar et al., 2014, p.4)  - 9.4 Misrepresent whole evidence bases:   - 9.4.1 Selective promotion of favourable parts of the evidence base: e.g. Coca-Cola producing an advert to divert attention to “energy balance” and away from overconsumption of sugary drinks (Nestle, 2015, p.238); Citing own (non-peer-reviewed) science to refute unfavourable peer reviewed evidence e.g. the tobacco industry citing its symposia proceedings as if they were balanced reviews of the literature in public relations materials, in attempts to create doubt about second-hand smoke (Bero, 2013, p.159) - 9.4.2 Deliberately overlooking unfavourable parts of the evidence base: e.g. the gambling industry calling evidence to support policy “meagre” (Thomas et al., 2018, p.23); e.g. third parties funded by the alcohol industry denying an association between alcohol consumption and breast cancer (Petticrew et al., 2017, p.5); e.g. the American Petroleum Institute attacking particular climate studies in a piecemeal way and ‘wilfully’ misunderstanding that the studies are part of a larger body of work (Union of Concerned Scientists, 2007, p.23); e.g. the vinyl chloride industry removing information on harms from a presentation to National Institute of Occupational Safety and Health saying “possible consumer safety and related potential hazards would not be pertinent to a presentation to NIOSH which is concerned with employee health matters” (White and Bero, 2010, p.119); a pharmaceuticals manufacturer was aware of adverse effects of interactions between two drugs, but waited eighteen months to include this in product information (Schott et al. 2010B, p. 299) - 9.4.3 Framing the evidence base or phenomenon as too complex for others to understand: e.g. the silica industry writing that silicosis is “far too complex, too involved and too little understood to be suited to mass-meeting methods” (White and Bero, 2010, p.119)   - 9.5 Misrepresent expert consensus: e.g. the Chlorine Institute releasing a press release announcing scientists had reached a consensus at their conference (which was convened jointly with the EPA) on the science around harms of dioxin, when consensus had not been reached (McGarity & Wagner, 2008, p.198). |
| 10. Monitor and attack scientists and organisations^[[11]](#footnote-11)^   \| Purpose: To weaken the influence of individuals and/or organisations that create or disseminate science deemed unfavourable by industry \| \| --- \|  \| Supporting evidence: (4, 6, 7, 10, 16, 17, 19, 20, 22, 25, 31, 32, 34, 37-39, 48, 53-55) \| \| --- \|  \| Tobacco \| Pharma \| \| --- \| --- \| \| Alcohol \| Fossil fuels \| \| Food and drink \| Extractive \| \| Chemicals/manufact. \| Gambling \| | - 10.1 Monitor the opposition in order to weaken it - 10.2 Attack individual scientists and whole cohorts of researchers - 10.3 Remove individual scientists from positions of power - 10.4 Attack organisations that create and disseminate science | - 10.1 Monitor the opposition in order to weaken it: e.g. the tobacco industry’s so-called “Project Sunrise” which involved compiling intelligence on individuals and organisations working in tobacco control, framing some as “extreme” and others as “moderate” in order to divide the community (McDaniel et al., 2006, p.217); e.g. PR firm Hill & Knowlton proposing a strategy to the beryllium industry for identifying and monitoring “groups hostile to beryllium” in order to later meet with them and “communicate the facts about beryllium, and dilute opposition group’s effects” (McGarity & Wagner, 2008, p.214)  - 10.2 Attack individual scientists and whole cohorts of researchers in order to intimidate them, undermine their reputations and/or drain their resources:   - 10.2.1 Attack individual scientists: e.g. the lead industry labelling an academic an ‘over-emotional, untrustworthy anti-lead fanatic’ (Union of Concerned Scientists, 2012, p.18) and filing a scientific misconduct complaint against him with the US government’s Office of Scientific Integrity (McGarity & Wagner, 2008, p.161); e.g. the tobacco industry launching an attack on Takeshi Hirayama’s reputation and his research on passive smoking (Pinto, 2017, p.59); e.g. the fossil fuels industry working through third party, the Global Climate Coalition, to attack scientist Benjamin Santer, an author on the 1996 IPCC report on climate change (Dunlap and McCright, 2011, p.150); suing researchers in order to silence them e.g. manufacturer of sleeping pill, Halcion, suing a scientist who had accused them of covering up unfavourable science (McGarity & Wagner, 2008, p,170) - 10.2.2 Attack whole cohorts of researchers e.g. tobacco companies labelling scientists investigating the health effects of smoking as the “anti-smoking industry” and describing their aims as to “manufacture alleged evidence, suggestive inferences linking smoking to various diseases and publicity and dissemination and advertising of these so-called findings to the widest possible public’” (Diethelm and McKee, 2009, p.2)   - 10.3 Remove independent scientists from positions of power: e.g. ExxonMobil successfully lobbying the Bush administration in order to remove the chair of the Intergovernmental Panel on Climate Change (IPCC) (Union of Concerned Scientists, 2007, p.20)  - 10.4 Attack organisations that create or disseminate science: e.g. The (Kraft Foods-funded) Tufts Navigator calling the Centre for Science in the Public Interest “sensationalist and alarmist” (Nestle, 2013, p.129); e.g. Philip Morris encouraging federal investigations of tobacco control organisations’ funding (McDaniel et al., 2006, p.218) |
| Macro Strategy C. Influence the reach of science to create an “echo chamber” for industry’s scientific messaging | | |
| 11. Use legal means to protect industry evidence from being discovered or accessed   \| Purpose: To limit corporations’ vulnerability to any scrutinisation including litigation around the harms of their products or practices \| \| --- \|  \| Supporting evidence: (4, 14, 16, 23, 35, 56) \| \| --- \|  \| Tobacco \| Pharma \| \| --- \| --- \| \| Alcohol \| Fossil fuels \| \| Food and drink \| Extractive \| \| Chemicals/manufact. \| Gambling \| | - 11.1 Prevent research from being undertaken in countries where corporations are vulnerable to litigation based on legal advice - 11.2 Limit internal communication to mask industry knowledge of harms based on legal advice - 11.3 Store scientific documents in ways which would prevent their discovery based on legal advice - 11.4 Use “proprietary information” claims when pressed by courts to release industry evidence - 11.5 Attempt to embed mechanisms in trade and investment treaties which prevent access to industry evidence - 11.6 Silence plaintiffs using secret payments | -11.1 Prevent research from being undertaken in countries where corporations are vulnerable to litigation based on legal advice: e.g. lawyers advising British American Tobacco (BAT) not to conduct research in countries such as Canada, Germany and Brazil which were seen as “places…helpful to plaintiffs” (Hanauer, 1995, p.238)  - 11.2 Limit internal communication to mask industry knowledge of the harms caused by its products or practices based on legal advice: e.g. BAT’s and Brown and Williamson’s (B&W) lawyers advised staff not to engage in ‘careless’ scientific discussion about health effects of smoking (Hanauer et al., 1995, p. 236), and BAT/B&W agreeing to limit discussion on smoking and health issues to in-person meetings instead of correspondence (Hanauer et al., 1995, p.237); e.g. the tobacco industry using the code-word “zephyr” for cancer in internal memos about research (Bero, 2013, p.161)  - 11.3 Store scientific documents in ways which would prevent their discovery based on legal advice: e.g. tobacco industry lawyers advising labelling scientific documents as “work product” or “privileged” to prevent them from being discovered and used in litigation (Hanauer, p.237); tobacco industry lawyers labelling certain scientific documents “deadwood” (these were mouse-skin painting studies that demonstrated the carcinogenicity of tobacco tar) and advising they be stored in a basement, and that employees should not make records of them (Hanauer et al., 1995, p. 238)  - 11.4 Use “proprietary information” claims when pressed by courts to release industry evidence: e.g. A mining corporation attempted to restrict access to information about the impacts of their mine by claiming in an *amicus curiae* brief (a document provided to courts) that data about river pollution that had been shared with US government officials was “proprietary business information” and was therefore legally protected from disclosure (Kirsch, 2014, p.149).  - 11.5 Attempt to embed mechanisms in trade and investment treaties which prevent access to industry evidence, meaning it cannot be scrutinised by the public, courts, experts and/or competitors: e.g. lobby groups CropLife America and the European Crop Protection Association (which represent pesticides corporations) used trade and investment treaty negotiations between the US and the EU on TTIP (the proposed Trans-Atlantic Trade and Investment Partnership) in attempts to implement “exclusive use periods” which would have blocked public access to data on product risks (Smith, E. et al., 2015, p.18). These groups also argued for the implementation of confidential business information (CBI) provisions within the treaty which would “shelter CBI data from public and scientific peer review” (Smith, E. et al., 2015, p.18). These particular efforts were ultimately unsuccessful because TTIP negotiations were halted.  - 11.6 Silence plaintiffs using secret payments: e.g. the manufacturer of Zomax (an arthritis medicine) knew its risks yet kept marketing it, and when it reached the courts, paid bonus payments to plaintiffs-victims to legally suppress the fact that the company knew that evidence on harms existed (McGarity and Wagner, 2008, p.122) |
| 12. Contract messengers to create scientific echo chambers^[[12]](#footnote-12)^   \| Purpose: To amplify and maximise the reach of industry scientific messages by channelling them through a multitude of trusted voices \| \| --- \|  \| Supporting evidence: (1-22, 25, 27, 28, 31-39, 41-46, 48-50, 52-54, 56-63) \| \| --- \|  \| Tobacco \| Pharma \| \| --- \| --- \| \| Alcohol \| Fossil fuels \| \| Food and drink \| Extractive \| \| Chemicals/manufact. \| Gambling \| | - 12.1 Create front groups to amplify industry-friendly scientific messages - 12.2 Fund third parties in order to amplify or shape their scientific stances - 12.3 Recruit, fund, and train individuals to be trusted scientific voices for industry - 12.4 Strategically create and fund a multitude of voices to manufacture a picture of scientific consensus - 12.5 Build industry coalitions (both within an industry, and with allied industries) to demonstrate support for industry-friendly scientific stances | - 12.1 Create front groups to amplify industry-friendly scientific messages: e.g. the Beverage Institute for Health and Wellness (BIHW), a front group founded by Coca-Cola who “uses this Institute to present its version of facts about nutrition and health to health professionals. Because position statements come from BIHW, not Coca-Cola itself, they appear to be issued by a legitimate unbiased source of scientific information” (Nestle, p.335); e.g. the Information Council on the Environment (ICE) which was set up by the fossil fuels industry to “reposition global warming as a theory (not fact)” (Dunlap and McCright, 2011, p.150).  - 12.2 Fund third parties in order to amplify or shape their scientific stances: e.g. the fossil fuels industry funding think tanks and foundations such as the American Enterprise Institute (AEI) and Cato Institute which disseminate climate denial science (Dunlap and McCright, 2011, p.147) and the industry-funded American Council on Science on Health which has defended industry scientific stances on issues concerning fossil fuels and chemicals (McGarity & Wagner, 2008, p.191); e.g. The Society for Women’s Health Research which became majority-funded by the pharmaceutical industry and supported the industry’s scientific stance on hormone replacement drugs (McGarity & Wagner, 2008, p.192); e.g. Coca-Cola funding professional bodies such as the American Academy of Pediatric Dentistry which subsequently changed its stance on sugar-sweetened beverages (Union of Concerned Scientists, 2012).  - 12.3 Recruit, fund and train individuals (such as scientists, medical professionals, and those working in government and other official positions) to be trusted scientific voices for industry: e.g. the pharmaceutical industry using academics in the media to promote cancer drug Herceptin (Shimazawa, 2014, p.494); e.g. PMI’s “Project Whitecoat” and the Tobacco Institute’s “Environmental Tobacco Smoke Academic Scientists Team”, both of which recruited scientists for public relations purposes, with the latter referred to as “foot soldiers” (McGarity & Wagner, 2008, p.193); using PR firms to train scientists e.g. the asbestos industry using PR firm Hill and Knowlton to train industry scientists to explain their scientific standpoints at symposia and in testimony (McGarity & Wagner, 2008, p,214); e.g. the tobacco industry recruiting a German scientist who was a member of government working groups on smoking where he spoke for the industry and “described smoking as a regularly satisfying experience…which perhaps might reduce workplace absenteeism” (Gruning et al., 2006, p. 23)  - 12.4 Strategically create and fund a multitude of voices to manufacture a picture of scientific consensus: e.g. the creation of a climate change denial machine, used to “distribute a flood of denial material” and “amplify the voices of contrarian scientists”, with one such body the Cooler Heads Coalition (CHC) (a US climate change denial front group which had think-tanks such as the Heartland, Marshall and Competitive Enterprise Institutes as members) functioning as a subgroup of the National Consumer Coalition, which was itself founded by industry-funded body “Consumer Alert” (Dunlap and McCright, 2011, p.151).  - 12.5 Build industry coalitions (both within an industry, and with allied industries): e.g. the alcohol industry working through one of its US trade associations the Distilled Spirits Council of the United States (DISCUS) to criticise independent science linking alcohol advertising to increased alcohol consumption (Babor, 2009, p.43); e.g. the tobacco industry attempting to mobilise “allied” industries to support its attempts to advance legislation on data access and data quality (“sound science” legislation)^[[13]](#footnote-13)^ (Bero, 2013, p.163) |
| 13. Fund, produce and disseminate materials which package science in industry-favourable ways^[[14]](#footnote-14)^     \| Purpose: To ensure industry-friendly scientific messages are disseminated widely beyond the scientific literature to key stakeholders \| \| --- \|  \| Supporting evidence: (1, 2, 4, 11, 14-19, 21, 22, 30-32, 35, 37-42, 44, 47, 50, 57-60, 64, 65){Mandrioli, 2016, Relationship between Research Outcomes and Risk of Bias`, Study Sponsorship`, and Author Financial Conflicts of Interest in Reviews of the Effects of Artificially Sweetened Beverages on Weight Outcomes: A Systematic Review of Reviews. } \| \| --- \|  \| Tobacco \| Pharma \| \| --- \| --- \| \| Alcohol \| Fossil fuels \| \| Food and drink \| Extractive \| \| Chemicals/manufact. \| Gambling \| | - 13.1 Fund, produce and disseminate lobbying materials that summarise science in industry-favourable ways - 13.2 Fund, produce and disseminate textbooks, letters to the editor, practice guidelines and other educational or academic materials - 13.3 Fund, produce and disseminate “easily-digestible” materials such as factsheets, newsletters, and product information materials - 13.4 Obtain and disseminate reprints of industry-favourable scientific outputs | - 13.1 Produce and disseminate lobbying materials (e.g. policy submissions, policy briefs, and “action kits”) that summarise science in industry-favourable ways: e.g. alcohol industry-funded policy submissions in Scotland which referred to the evidence base around minimum unit pricing of alcohol in misleading ways (Cullen et al., 2017, p.1); policy briefs e.g. the International Centre for Alcohol Policies (ICAP) producing ‘Drinking in Context: Patterns, Interventions and Partnerships’ which was disseminated to policymakers in LMICs for use as a policy brief, and has been “widely criticised for misrepresenting the public health view on alcohol policies” (Babor and Robaina, 2013, p. 209); “action kits” e.g. the International Tobacco Information Centre (INFOTAB), a group set up by tobacco manufacturing associations to “maintain a common front on scientific and political issues” produced “action kits” to assist its members in their lobbying efforts (Lee et al., 2012, p.124)  - 13.2 Produce and disseminate textbooks, letters to the editor, practice guidelines and other “educational” or academic material for students, academics, health professionals and the public: e.g. pharmaceuticals industry funding and disseminating educational textbooks (Stamatakis et al., 2013, p.472); e.g. up to 87% of clinical practice guideline authors have conflicts of interest with the pharmaceutical industry, and such materials are “often heavily focused on new costly interventions and only loosely follow the available evidence” (Stamatakis et al., 2013, p.472); e.g. the alcohol industry-funded International Life Sciences Institute (ILSI) producing and disseminating the book “Health Issues Related to Alcohol Consumption”, which made no mention of its industry funding (Babor, 2009, p.40)  - 13.3 Produce and disseminate “easily-digestible” materials such as factsheets, newsletters and product information materials: e.g. the American Dietetic Association publishing factsheets funded and written by industry such as “Adult Beverage Consumption: Making Responsible Drinking Choices” sponsored by the Distilled Spirits Council (Brownell and Warner, 2009, p.277); e.g. newsletters such as the tobacco industry-funded monthly newsletter from the TIRC, “The Tobacco and Health Report” (Pinto, 2017); e.g. industry-funded product information materials on breast-milk substitutes disseminated to patients and health professionals in West Africa which misrepresented the evidence base on the hazards of using such products (Grundy et al., 2013, p.5)  - 13.4 Obtain and disseminate reprints of industry-favourable scientific publications: e.g. Philip Morris, as a pre-emptive strike against the EPA risk assessment on second-hand smoke, sending a favourable output (“the Yale study”) to over 300 scientists (McGarity & Wagner, 2008, p.206); pharmaceuticals company Merck, distributing industry-favourable science from its own journal, the Australian Journal of Bone and Joint medicine, to 20,000 doctors (Union of Concerned Scientists, 2012, p.16). |
| 14. Use education, events, and meetings to disseminate industry-favourable scientific messages to key stakeholders   \| Purpose: To ensure industry-friendly scientific messages are disseminated directly to key stakeholders including students, health professionals, academics, policymakers and the public \| \| --- \|  \| Supporting evidence: (1-5, 7-11, 16-19, 21, 22, 28, 30-35, 38, 39, 42, 46, 47, 49, 53, 57, 58, 60-62, 64-66) \| \| --- \|  \| Tobacco \| Pharma \| \| --- \| --- \| \| Alcohol \| Fossil fuels \| \| Food and drink \| Extractive \| \| Chemicals/manufact. \| Gambling \| | - 14.1 Fund, organise and speak at ‘educational events’ for key stakeholders - 14.2 Access policymakers through meetings and hearings - 14.3 Infiltrate decision-making contexts in order to ensure industry-friendly scientific stances are heard - 14.4 Train and use industry representatives to meet with health professionals | - 14.1 Fund, organise and present at “educational events” for key stakeholders (e.g. students, health professionals, academics, policymakers and the public):   - 14.1.1 Fund and organise scientific conferences and  meetings: e.g. Brush Wellman (a beryllium manufacturer) sponsoring a conference in collaboration with the American Conference of Governmental Industrial Hygienists to promote the corporation’s evidence on exposure standards (Michaels, 2008, p.95); e.g. the tobacco industry sponsoring and organising conferences and funding scientists to attend them in order to disseminate industry messages on the science around second-hand smoke (Bero, 2013, p.159); e.g. the alcohol industry funding a symposia at the annual meeting of the Research Society on Alcoholism which “explored the relationship between the private sector and the research community” (Babor, 2009, p.42) - 14.1.2 Fund and organise formal education for students and health professionals: e.g. the pharmaceutical industry sponsoring continuing medical education (CME), of which some “preferentially highlighted the sponsor’s drugs compared with other CME programs” (Shimazawa &Ikeda, 2014, p.492); Coca-Cola funding and providing education for dieticians and nurses (Nestle, p.256) - 14.1.3 Fund and organise education for the public e.g. Coca-Cola funding nutrition education for the public in Chicago (Nestle, Soda Politics, p.238); e.g. the American Enterprise Institute holding a public lecture with popular author, Michael Crichton entitled “Science Policy in the Twenty-First Century” where Crichton called the science on global warming “shockingly flawed and unsubstantiated” (McGarity & Wagner, 2008, p.147)     -14.2 Access policymakers through meetings and hearings: e.g. the silica industry repeatedly meeting with officials about safety standards (Union of Concerned Scientists, 2012, p.23); presenting evidence at official hearings e.g. the Lead Industry Association speaking at a subcommittee on pollution to dispute evidence about lead hazards (White and Bero, 2010, p.128); e.g. oil and rubber industry representatives putting pressure on officials at the International Agency for Research on Cancer (IARC) meetings to change butadiene classifications (Sass, 2005)    - 14.3 Infiltrate decision-making contexts in order to ensure industry-friendly scientific stances are heard e.g. members of the Lead Industry Association serving as Chair and Member of the American Standards Association Committee on Toxic Dusts and Gases and working to change safety standards (White and Bero, 2010, p.124)    - 14.4 Train and use industry representatives to meet with health professionals: using ‘detail men’ (pharmaceutical industry sales representatives) to make personal visits to health professionals training them to “tactfully avoid physician questions on safety” concerning certain drugs such as Vioxx (Stamatakis et al., 2013, p.471). |
| 15. Maximise press coverage of industry-favourable scientific messages^[[15]](#footnote-15)^   \| Purpose: To influence the ways in which science concerning industry products/practices/regulation is depicted in the media \| \| --- \|  \| Supporting evidence: (2-4, 6, 7, 14-17, 19-22, 24, 27, 31, 32, 34, 35, 37-39, 41, 42, 46, 49, 55) \| \| --- \|  \| Tobacco \| Pharma \| \| --- \| --- \| \| Alcohol \| Fossil fuels \| \| Food and drink \| Extractive \| \| Chemicals/manufact. \| Gambling \| | - 15.1 Create favourable media content - 15.2 Fund media outlets in order to influence what is disseminated - 15.3 Co-opt journalists through media training and conference funding      - 15.4 Design events intended to maximise media coverage - 15.5 Demand media coverage of industry scientific arguments in the name of ‘balance’ | - 15.1 Create media content such as press releases, op-eds, advertisements, public service announcements: e.g. Coca-Cola taking out advertisements to emphasise the link between exercising and burning calories (from their products) (Nestle, 2015, p.238); e.g. The Society of the Plastics Industry publishing a press release to critique a study linking miscarriage to vinyl chloride (White and Bero, 2010); e.g. the Western Fuels Association spending $250,000 on creating a video for use in the media which promoted the supposed benefits of global warming (McGarity & Wagner, 2008, p.216)  - 15.2 Fund media outlets in order to influence what is disseminated: e.g. ExxonMobil funding Tech Central Station website (Union of Concerned Scientists, 2007, p.13)  - 15.3 Co-opt journalists through media training and conference funding: e.g. a PR company working with the American Petroleum Institute on its Global Climate Science Communication Action Plan providing media training for journalists (McGarity & Wagner, 2008, p.212); e.g. The Chlorine Chemical Council and its thirty-city tour, sending scientists to meet with journalists to talk about dioxin (McGarity & Wagner, 2008, p.217); e.g. a Coca-Cola funded think-tank organising a conference, on “Sweetness, Sugar and Health” and paying journalists to attend (McGarity & Wagner, 2008, p.198).  - 15.4 Design events intended to maximise favourable media coverage: e.g. Philip Morris launching The Advancement of Sound Science Coalition away from “cynical reporters from major media” (Union of Concerned Scientists, 2007, p.18); e.g. Hill & Knowlton working with the dioxin industry to organise a symposium described as a “situation designed for maximum media exposure” (McGarity & Wagner, 2008, p.215)  - 15.5 Demand media coverage in the name of “balance” e.g. tobacco industry lawyers exploiting the idea of “balanced” press coverage by demanding equal time for both sides of the “debate” on the health harms of smoking (Pinto, 2017, p.58) |
| Macro Strategy D. Create industry-friendly policymaking environments which shape the use of science in policy decision-making in industry’s favour | | |
| 16. Implement and utilise industry-friendly standards of evidence in regulatory decision-making     \| Purpose: To reduce policymakers’ ability to use unfavourable evidence in official assessments of risk and their ability to use a precautionary approach to regulation \| \| --- \|  \| Supporting evidence: (4, 16, 44-46, 49, 56, 62, 67) \| \| --- \|  \| Tobacco \| Pharma \| \| --- \| --- \| \| Alcohol \| Fossil fuels \| \| Food and drink \| Extractive \| \| Chemicals/manufact. \| Gambling \| | - 16.1 Attempt to implement industry-friendly standards of evidence which set a high evidential bar within regulatory decision-making - 16.2 Utilise such standards in attempts to undermine the use of unfavourable science in regulatory decision-making | - 16.1 Attempt to implement industry-friendly standards of evidence which set a high evidential bar within regulatory decision-making: Such efforts, which have varied in effectiveness, have focused largely on promoting the use of risk assessments which set the evidential bar high enough to dismiss evidence the industry might deem unfavourable and, more broadly, promote a risk-based rather than a precautionary-based approach to policy making. e.g. Philip Morris and others (including the chemicals industry) lobbied to embed standards that evidence would have to meet before it could be officially considered by policy makers as part of EU regulatory mechanisms (standards they had previously proposed and promoted for the creation and interpretation of “good epidemiology” – strategy 7.1); these particular efforts did not result in any such mandatory standards) (Ong and Glantz, 2001; Smith et al. IN Bero, 2013, p.164); e.g. British American Tobacco (from 1995 onwards) attempted to secure an amendment to the EU treaty which would implement “structured risk assessment” within the EU “Better Regulation” agenda. This was intended to have strict “rules for the assessment of epidemiological and animal data” that could “remove the possibility of introducing public smoking restrictions that are based on risk claims” (Smith et al., IN Bero, 2013, p.164); e.g. more generally, pesticides lobby groups used trade and investment treaty negotiations to push for a structured toxicological risk assessment approach to the evaluation of endocrine-disrupting chemicals (naming this “science-based” regulation). This was part of attempts to lower EU standards of protection against pesticides to US levels, which industry euphemistically calls “harmonization” (Smith, E., et al., 2015). This was despite experts concluding that, due to limits on current research methodologies, it would be nearly impossible to accurately determine a safe level of exposure to endocrine disruptors, which have been shown to break “all the rules and assumptions that have guided toxicology through the era of modern chemical regulation.”(ibid). This so-called “science-based” regulation has been described as a “veiled and publicly palatable way through which to attack the precautionary principle” (Lydgate et al., 2020, p.5); e.g. The tobacco industry had success embedding industry-friendly scientific standards in US policymaking environments, where Philip Morris and others (in response to the EPA risk assessment on second-hand smoke) lobbied for (and secured) the implementation of the US Information Quality Act (IQA - also known as the Data Quality Act). This Act required government agencies to produce quality guidelines for the science used in their decision-making, rather than relying on peer-review as a quality standard for science, as was previously the case. It also embedded a mechanism through which third parties (including industry) could challenge the science used, on the basis of these quality standards (often in ways outlined in strategy 9) (Baba et al., 2005; McGarity and Wagner, 2008, p.155-156.(16)  - 16.2 Utilise these regulatory mechanisms and tools in order to undermine the use of unfavourable science in regulatory decision-making: e.g. In the five years after its implementation, the IQA was used by the chemicals, food and fossil fuels industries to challenge and dismantle evidence bases unfavourable to their interests (McGarity and Wagner, 2008, p,151). They did so by challenging minor aspects of many individual studies which collectively enabled conclusions about the risks of regulated products, using this approach “to undermine…often well-accepted weight-of-the-evidence risk judgements.” (ibid) For example, when the National Toxicology Programme (NTP) advised putting industry solvent trichloroethylene (TCR) on a list of human carcinogens, The Halogenated Solvents Industry Alliance, enabled by the IQA, attempted to discredit each of the epidemiological and animal studies upon which the decision was based ultimately pushing the NTP not to place TCR on its carcinogens list.(McGarity and Wagner, 2008, p. 155-156). |
| 17. Secure and utilise policymaking reforms which increase reliance on and provide a conduit for industry-favourable evidence   \| Purpose: To maximise industry’s ability to use favourable science to influence policy decisions \| \| --- \|  \| Supporting evidence: (1, 4, 53, 56, 59-63, 67) \| \| --- \|  \| Tobacco \| Pharma \| \| --- \| --- \| \| Alcohol \| Fossil fuels \| \| Food and drink \| Extractive \| \| Chemicals/manufact. \| Gambling \| | - 17.1 Secure the implementation and use of mandatory regulatory tools such as business impact assessments which increase reliance on industry data and prioritise evidence on economic impacts - 17.2 Secure and utilise changes to upstream policymaking architecture which embed industry’s right to participate in policymaking processes^[[16]](#footnote-16)^ - 17.3 Secure reductions in evidence requirements for some regulatory decision-making to maximise the use of industry-favourable evidence | - 17.1 Secure the implementation and use of mandatory regulatory tools such as business impact assessments which create a reliance on industry data and prioritise evidence on economic impacts (over other impacts such as human and environmental health):   - 17.1.1 Work to secure these changes: e.g. as part of British American Tobacco’s campaign (with other sectors of industry including chemical, fossil fuels and pharmaceuticals) for regulatory reforms in the EU (which became known as “Better Regulation”), from the mid-1990s onwards it worked to mandate the use of business impact assessment using a Cost Benefit Analysis (CBA) approach (alongside stakeholder consultation – see strategy 17.2) as a means for assessing the impacts of a proposed policy (Smith et al. 2010a). These impact assessments would focus heavily on business and economic impacts, meaning that all EU policy decision-making would be made within an economic framework (Smoke Free Partnership, 2010) and that “reductions in risks to human health (and the environment) would be partially assessed on the basis of their economic efficiency” (Smith et al. 2010b, p.4), requiring “non-market goods” which are difficult to quantify “to be assessed in monetary terms” (Smoke Free Partnership, 2010, p.12). Legal firm Ernst & Young advised British American Tobacco to campaign for these regulatory reforms as the use of cost benefit analysis (CBA) within impact assessments would mean that “greater attention and credibility will be…[given]…to the industry-developed statistical series. This can be used to advantage in discussions and negotiations with government agencies as it means that the industry has access to information…that are unavailable to government officials” (Smith, K. et al., 2015, p.343) - 17.1.2 Use these tools in ways that support industry interests: e.g. in response to an official draft regulatory impact assessment related to the EU’s 2001 Tobacco Product’s Directive, the Tobacco Manufacturers’ Association argued that the costs to business would actually be higher, “providing alternative (higher) compliance costs” (Smith et al, 2010b), and British American Tobacco produced a report which “overplayed the potential job losses (and related economic costs) associated” with the proposed legislation (ibid); e.g. lobby groups for pesticides corporations produced their own impact assessment on the potential effects of bans on some endocrine disrupting chemicals in the EU and exaggerated the financial effect such regulation would have on international trade. To produce this “highly exaggerated figure, CropLife and ECPA ignored existing alternatives…and assumed worst-case, unrealistic estimates for yield losses” (Smith, E. et al., 2015; p. 16)   17.2 Secure and utilise changes to upstream policymaking architecture which embed industry’s right to participate in policymaking processes:   - 17.2.1 British American Tobacco (as part of efforts described above to mandate the use of business impact assessments in EU policymaking - see 17.1, which would become known as “Better Regulation” regulatory reform) also lobbied for mandatory stakeholder consultations (Smith et al., 2010).While ostensibly about transparency and good governance, in actuality this embedded industry’s right to be formally consulted early on in policymaking processes and therefore to have their scientific viewpoint heard (Smoke Free Partnership, 2009, p. 22); pesticides lobby groups similarly worked to embed mandatory stakeholder consultation in trade and investment treaty provisions for these same reasons (Smith, E., et al., 2015, p5) - 17.2.2 Utilise these changes in ways that support industry interests: e.g. in the UK policy debate on plain packaging of cigarettes, the tobacco industry used stakeholder consultation to overwhelm the consultation with submissions, within which the industry misrepresented the evidence base on plain packaging,^[[17]](#footnote-17)^ (Ulucanlar et al., 2014, p.1). The authors of this article critique stakeholder consultation as a tool which provides “an opportunity for highly resourced corporations to slow, weaken, or prevent public health policies” (Ulucanlar et al., 2014, p.1)   - 17.3 Secure reductions in evidence requirements for some regulatory decision-making to maximise the use of industry-favourable evidence by:   - 17.3.1 Securing reductions in safety data requirements for drug approval processes: e.g. the pharmaceutical industry worked through the International Conference on Harmonisation of Technical Requirements for Registration of Pharmaceuticals for Human Use (ICH) to shorten the duration of post-trial carcinogenicity testing required for pharmaceutical approval (Abraham, 2002, p.1500) - 17.3.2 Using trade and investment treaties to reduce the safety thresholds required in regulatory decision-making: e.g. the pesticides industry attempted to use TTIP (the proposed Trans-Atlantic Trade and Investment Partnership) to lower EU food safety standards to bring them in line with lower US standards by (a) requiring the EU to accept data from US trials (rather than using its own, more stringent trial data and (b) embedding the practice of “extrapolation” – that is, “permitting data from two or three representative crops” to be used for setting the maximum residue levels for related crops “that have not undergone trial tests” (Smith. E., et al., 2015, p.13). Such a practice “places consumers at increased risk of harm” because residual pesticides levels may be different for different crops and so representative crop trials are needed. (ibid). |
| Macro Strategy E. Manufacture trust in industry and its scientific messaging (in turn enabling the entire scientific strategy) | | |
| 18. Manufacture a picture of industry credibility   \| Purpose: To create legitimacy around industry and industry science, and to frame industry as an appropriate and even essential partner in science and scientific decision-making \| \| --- \|  \| Supporting evidence: (1-4, 6-11, 13-17, 19-22, 25, 30-35, 37-39, 41, 42, 47, 51, 58, 68) \| \| --- \|  \| Tobacco \| Pharma \| \| --- \| --- \| \| Alcohol \| Fossil fuels \| \| Food and drink \| Extractive \| \| Chemicals/manufact. \| Gambling \| | - 18.1 Ensure and normalise industry’s presence in academic settings in attempts to gain trust and scientific credibility within academia^[[18]](#footnote-18)^ - 18.2 Promote industry’s overt links with expert individuals and organisations to manufacture a picture of industry scientific credibility more broadly - 18.3 Inflate the scientific credibility of industry science, scientists, and scientific perspectives | - 18.1 Ensure industry’s presence in academic settings in attempts to gain trust and perceived credibility *within* academia:   - 18.1.1 Fund academics (e.g. researchers, peer-reviewers, editors, authors of clinical practice guidelines) through e.g. grants, honoraria, donations, awards, consulting fees, stocks etc. (Nestle, 2015, p.265; Stamatakis et al., 2013, p.471) - 18.1.2 Fund students (through e.g. educational scholarships, lunches, gifts etc.): e.g. the tobacco industry creating scholarships to fund medical students (Pinto, 2017, p.57); e.g. Diageo funding students at Geary University College Dublin (Babor, 2009, p.38); e.g. undergraduate medical students in receipt of industry-sponsored gifts (Stamatakis et al., 2013) - 18.1.3 Fund academic infrastructure e.g. research centres: e.g. BP paying $500m to the universities of Illinois and California-Berkeley to create the Energy Biosciences Institute (McGarity & Wagner, 2008, p.255)   - 18.2 Promote industry’s overt links with expert individuals and organisations in order to manufacture a picture of industry scientific credibility more broadly e.g.   - 18.2.1 Use recruitment and funding of scientific experts to enhance industry credibility: e.g. the tobacco industry recruiting renowned scientist Clarence Cook Little to head up the Tobacco Industry Research Committee, (Pinto, 2017, p.61) and Fred Seitz working for RJ Reynolds, who then “gained the credentials of one of the most respected scientists in the country” (Pinto, 2017, p.58); e.g. the gambling industry wanting to get “kudos” for working with academics (Cassidy et al., 2013); - 18.2.2 Use funding of research institutions and public health/professional organisations in order to enhance industry credibility: e.g. energy companies funding the $225m Global Climate and Energy Project at Stanford University, after which Exxon Mobil advertised its relationship with the “best minds” and spoke of “lively debate” about greenhouse gases and climate change (McGarity & Wagner, 2008, p.252); e.g. Coca-Cola becoming corporate partner of American Dietetic Association (ADA), providing Coca-Cola “a national platform via ADA events and programs with prominent access to key influencers, thought leaders and decision makers in the nutrition marketplace” (Brownell and Warner, 2009, p.277)   - 18.3 Inflate the credibility of industry science, scientists and scientific perspectives:   - 18.3.1 Overstating the rigor of industry scientific outputs: e.g. the Australian Wine Research Institute claiming to have “published many papers” on alcohol and health, where the majority they listed were speeches and articles in trade journals (Babor, 2009, p.41); e.g. the tobacco industry citing its own symposia articles as “the consensus of a gathering of ‘leading experts from around the world’ who disagree with the published literature on passive smoking” (Bero, 2013, p.159) - 18.3.2 Hyperbolising the credibility of industry scientists: e.g. in submissions to the UK government on plain packaging, the tobacco industry presented scientists as “the best contemporary scientific thinking” and focused on establishing the credibility of the scientists, with author CVs in one submissions “taking up 61 pages of a 98-page report” (Ulucanlar et al., 2014, p.7) - 18.3.3 Falsifying expert support for scientific messages: e.g. the Oregon Petition, a list of 17,000 signatures of supposed experts who rejected anthropogenic climate change (it transpired many names were fictional/had no expertise) (Dunlap & McCright, 2010) |
| 19. Conceal industry’s involvement in science, scientific messaging and influence on policy reforms which affect the use of science   \| Purpose: To create a false picture of independence (and therefore reputability) around some industry science and scientific messaging \| \| --- \|  \| Supporting evidence: (1, 3-6, 8-10, 12-16, 19, 20, 22, 24-26, 28-31, 33, 35-42, 44-46, 49, 57, 59-62, 68) \| \| --- \|  \| Tobacco \| Pharma \| \| --- \| --- \| \| Alcohol \| Fossil fuels \| \| Food and drink \| Extractive \| \| Chemicals/manufact. \| Gambling \| | - 19.1 Conceal industry funding of science, production of science and recruitment of scientists - 19.2 Conceal industry dissemination of science and scientific messages^[[19]](#footnote-19)^ - 19.3 Conceal industry attempts to shape ways in which science is used in policymaking | - 19.1 Conceal funding of science, production of science and recruitment of scientists:   - 19.1.1 Create or fund third party organisations (often with names which sound independent) to produce science: e.g. the Philip Morris creating the Institute for Biological research (Gruning et al., 2006, p.21); e.g. the Center for Indoor Air Research producing favourable research for the tobacco industry (Pinto, 2017, p.57); e.g. the tobacco industry covertly sponsoring symposia through third parties so that any proceedings would look independent (Bero, 2013, p.159) - 19.1.2 Create bodies within industry-funded organisations, such as scientific advisory boards, which give the illusion of a protective “firewall” from industry influence: e.g. the scientific advisory board of the Tobacco Industry Research Council (Brandt, 2012, p.67) - 19.1.3 Conceal recruitment of scientists: e.g. the tobacco industry using Covington and Burling law firm to recruit experts to refute harms of passive smoking (Lee et al., 2012, p.124); the pharmaceutical industry recruiting scientists through the American Enterprise Institute (Pinto, 2017, p.64) - 19.1.4 Fail to properly disclose industry involvement in science at publication stage: e.g. the pharmaceutical industry ghost-writing studies (or using ghost-writing companies to do so) and then publishing the science using prestigious “guest authors” such as the paper “Gabapentin for Treatment of Pain and Tremor” which was funded by pharmaceuticals company, Parke-Davis, but published with no disclosure of funding source (White and Bero, 2010, p. 114); e.g. research funded by the tobacco industry stating it had received “financial support” from tobacco companies, but not being transparent about the extent of industry-funded lawyers’ involvement in study design, analysis and reporting (Bero, 2013, p.158)   - 19.2 Concealing dissemination of industry-friendly science and scientific messages: e.g. the tobacco industry using third party organisation Cooperation Centre for Scientific Research Relative to Tobacco (CORESTA) to disseminate research findings and recommendations for tobacco product standards to the International Organization for Standardization (ISO) (Bialous and Yach, 2001); e.g. tobacco industry-funding letters to the editor which often do not disclose their financial conflicts of interest (Bero, 2013, p.162)  - 19.3 Concealing attempts to shape ways in which science is used in policymaking: e.g. BAT using the European Policy centre to conceal its attempts to mandate the use of business impact assessments in EU policymaking (Smith et al., 2010a, p.482) |

**References for S3.**

1. Akl EA, Khamis AM. The intersections of industry with the health research enterprise. Health Research Policy and Systems. 2019;17(53).

2. Babor TF, Robaina K. Public Health, Academic Medicine, and the Alcohol Industry's Corporate Social Responsibility Activities. American Journal of Public Health. 2013;103(2):206-14.

3. Babor TF. Alcohol research and the alcoholic beverage industry: issues, concerns and conflicts of interest. Addiction. 2009;104:34-47.

4. Bero LA. Tobacco Industry Manipulation of Research. Late Lessons from Early Warnings: Science, Precaution, Innovation EA report number 1/2013: European Environmental Agency; 2013.

5. Bialous SA, Glantz SA. ASHRAE Standard 62: tobacco industry's influence over national ventilation standards. Tob Control. 2002;11(4):315-28.

6. Brandt AM. Inventing Conflicts of Interest: A History of Tobacco Industry Tactics. American Journal of Public Health. 2012;102(1):63-71.

7. Cassidy R, Loussouarn C, Pisac A. Fair Game: Producing gambling research. Goldsmiths; 2013.

8. Fabbri A, Lai A, Grundy Q, Bero L. The influence of industry sponsorship on the research agenda: a scoping review. American Journal of Public Health. 2018;108(11).

9. Granheim SI, Engelhardt K, Rundall P, Bialous S, Iellamo A, Margetts B. Interference in public health policy: examples of how the baby food industry uses tobacco industry tactics. World Nutrition. 2017;8(2).

10. Gruning T, Gilmore A, McKee M. Tobacco Industry Influence on Science and Scientists in Germany. American Journal of Public Health. 2006;96(1):20-32.

11. Jernigan DH. Global Alcohol Producers, Science and Policy: the Case of the International Center for Alcohol Policies. American Journal of Public Health. 2012;102(1).

12. Kearns CE, Schmidt LA, Glantz SA. Sugar Industry and Coronary Heart Disease Research A Historical Analysis of Internal Industry Documents. Jama Internal Medicine. 2016;176(11):1680-5.

13. Kearns CE, Glantz SA, Schmidt LA. Sugar Industry Influence on the Scientific Agenda of the National Institute of Dental Research's 1971 National Caries Program: A Historical Analysis of Internal Documents. Plos Medicine. 2015;12(3).

14. Kirsch S. Mining Capitalism. The Relationship between Corporations and their Critics. Oakland, California: University of California Press; 2014.

15. Lee S, Ling PM, Glantz SA. The vector of the tobacco epidemic: tobacco industry practices in low and middle-income countries. Cancer causes & control : CCC. 2012;23 Suppl 1:117-29.

16. McGarity TO, Wagner, W.E. Bending Science: how special interests corrupt public health research. MA, USA: Harvard University Press; 2008.

17. Nestle M. Soda Politics: Taking on Big Soda (and winning). Oxford: Oxford University Press; 2015.

18. Petticrew M, Katikireddi SV, Knai C, Cassidy R, Maani Hessari N, Thomas J, et al. 'Nothing can be done until everything is done': the use of complexity arguments by food, beverage, alcohol and gambling industries. Journal of Epidemiology and Community Health. 2017;71(11).

19. Pinto MF. To Know or Better Not to: Agnotology and the Social Construction of Ignorance in Commercially Driven Research. Science and Technology Studies. 2017;30(2):53-72.

20. Smith KE, Savell E, Gilmore AB. What is known about tobacco industry efforts to influence tobacco tax? A systematic review of empirical studies. Tobacco Control. 2013;2.

21. Thomas S, Randle M, Pitt H, Bestman A, Daube M, Pettigrew S. A Public Health Framework for Assessing Gambling Industry Strategies and Tactics. Melbourne: Victorian Responsible Gambling Foundation; 2018.

22. Union of Concerned Scientists. Smoke, Mirrors and Hot Air. How Exxon Mobil uses Big Tobacco's tactics to manufacture uncertainty on climate science. 2007.

23. Hanauer P, Slade J, Barnes DE, Bero L, Glantz SA. Lawyer control of internal scientific research to protect against products liability lawsuits. The Brown and Williamson documents

1. JAMA. 1995;274(3):234-40.

24. Schott G, Pachl H, Limbach U, Gundert-Remy U, Lieb K, Ludwig W-D. The Financing of Drug Trials by Pharmaceutical Companies and Its Consequences Part 2: A Qualitative, Systematic Review of the Literature on Possible Influences on Authorship, Access to Trial Data, and Trial Registration and Publication. Deutsches Arzteblatt International. 2010;107(17):295-U13.

25. Capps B. Can a good tree bring forth evil fruit? The funding of medical research by industry. British Medical Bulletin. 2016;118(1):5-15.

26. Egilman DS, Billings MA. Abuse of epidemiology: Automobile manufacturers manufacture a defense to asbestos liability. International Journal of Occupational and Environmental Health. 2005;11(4):360-71.

27. Michaels D. Manufactured Uncertainty: Contested Science and the Protection of the Public's Health and Environment. Agnotology: the making and unmaking of ignorance. Stanford: Stanford University Press; 2008.

28. Moodie R, Stuckler D, Monteiro C, Sheron N, Neal B, Thamarangsi T, et al. Profits and pandemics: prevention of harmful effects of tobacco, alcohol, and ultra-processed food and drink industries. Lancet. 2013;381(9867):670-9.

29. Schott G, Pachl H, Limbach U, Gundert-Remy U, Ludwig W-D, Lieb K. The Financing of Drug Trials by Pharmaceutical Companies and Its Consequences Part 1: A Qualitative, Systematic Review of the Literature on Possible Influences on the Findings, Protocols, and Quality of Drug Trials. Deutsches Arzteblatt International. 2010;107(16):279-U14.

30. Stamatakis E, Weiler R, Ioannidis JPA. Undue industry influences that distort healthcare research, strategy, expenditure and practice: a review. European Journal of Clinical Investigation. 2013;43(5):469-75.

31. Union of Concerned Scientists. Heads they Win, Tails we Lose. How Corporations Corrupt Science at the Public's Expense. Union of Concerned Scientists; 2012.

32. Brownell KD, Warner KE. The Perils of Ignoring History: Big Tobacco Played Dirty and Millions Died. How similar is Big Food? The Milbank Quarterly. 2009;87(1).

33. Livingstone C. A case for clean conferences in gambling research. Drug and Alcohol Review. 2018;37:683-6.

34. McDaniel PA, Smith EA, Malone RE. Philip Morris’s Project Sunrise: weakening tobacco control by working with it. Tobacco Control. 2006;15(3):215-23.

35. White J, Bero LA. Corporate Manipulation of Research: Strategies are Similar across Five Industries. Stanford Law and Policy Review. 2010;21:105-34.

36. Andermann A, Pang T, Newton JN, Davis A, Panisset U. Evidence for Health II: Overcoming barriers to using evidence in policy and practice. Health Research Policy and Systems. 2016;14.

37. Dunlap RE, McCright AM. Climate change denial: sources, actors and strategies. Routledge Handbook of Climate Change and Society. Abingdon: Routledge; 2010. p. 240-59.

38. Dunlap RE, McCright AM. Organized climate change denial. The Oxford Handbook of Climate Change and Society. Oxford: Oxford University Press; 2011. p. 145-60.

39. Nestle M. Food politics: how the food industry influences nutrition and health. California: University of California Press; 2013.

40. Savell E, Gilmore AB, Fooks G. How does the tobacco industry attempt to influence marketing regulations? A systematic review. PLoS One. 2014;9(2):e87389.

41. Savell E, Fooks G, Gilmore AB. How does the alcohol industry attempt to influence marketing regulations? A systematic review. Addiction. 2015;111:18-32.

42. Shimazawa R, Ikeda M. Conflicts of interest in psychiatry: Strategies to cultivate literacy in daily practice. Psychiatry and Clinical Neurosciences. 2014;68(7):489-97.

43. Egilman DS, Bohme SR. Over a barrel: Corporate corruption of science and its effects on workers and the environment. International Journal of Occupational and Environmental Health. 2005;11(4):331-7.

44. Bingham E, Boden L, Clapp R, Hoppin P, Krimsky S, Michaels D, et al. Daubert: The Most Influential Supreme Court Ruling You've Never Heard Of. Boston: Tellus Institute; 2003.

45. Michaels D, Monforton C. Manufacturing Uncertainty: Contested Science and the Protection of the Public's Health and Environment. American Journal of Public Health. 2005;95.

46. Ong EK, Glantz SA. Constructing "sound science" and "good epidemiology": tobacco, lawyers, and public relations firms. Am J Public Health. 2001;91(11):1749-57.

47. Ulucanlar S, Fooka GJ, Hatchard JL, Gilmore AB. Representation and misrepresentation of scientific evidence in contemporary tobacco regulation: a review of tobacco industry submissions to the UK Government consultation on standardised packaging. PLoS Medicine. 2014;11(3):??check??

48. Diethelm P, McKee M. Denialism: what is it and how should scientists respond? European Journal of Public Health. 2009;19:2-4.

49. Baba A, Cook DM, McGarity TO, Bero LA. Legislating "sound science": The role of the tobacco industry. American Journal of Public Health. 2005;95:S20-S7.

50. Cullen D, Smith K, Collin J. 'Half-cut' science: a qualitative examination of alcohol industry actors' use of peer-reviewed evidence in policy submissions on Minimum Unit Pricing. Evidence and Policy. 2017.

51. Cullerton K, Donnet T, Lee A, Gallegos D. Playing the policy game: a review of the barriers to and enablers of nutrition policy change. Public Health Nutrition. 2016;19(14):2643-53.

52. Petticrew M, Maani Hessari N, Knai C, Weiderpass E. How alcohol industry organisations mislead the public about alcohol and cancer. Drug and Alcohol Review. 2017;DOI: 10.1111/dar.12596.

53. Abraham J. The pharmaceutical industry as a political player. The Lancet. 2002;360:1498-502.

54. Bjornberg KE, Karlson M, Gilek M, Hansson SE. Climate and environmental science denial: a review of the scientific literature published in 1990-2015. Journal of Cleaner Production. 2017;167:229-41.

55. Connor J, Kypri K. The alcohol industry, the government and the alleged defamation of public health advocates: A New Zealand Case Study. Drug and Alcohol Review. 2018.

56. Smith E, Azoulay D, Tuncak B. Lowest common denominator. How the proposed EU-US trade deal threatens to lower standards of protection from toxic pesticides.: Center for International Environmental Law; 2015.

57. Bialous SA, Yach D. Whose standard is it anyway? How the tobacco industry determines the International Organization for Standardization (ISO) standards for tobacco and tobacco products. Tobacco Control. 2001;10:96-104.

58. Rothman DJ, McDonald WJ, Berkowitz CD, Chimonas SC, DeAngelis CD, Hale RW, et al. Professional medical associations and their relationships with industry: a proposal for controlling conflict of interest. JAMA. 2009;301(13).

59. Smith KE, Fooks G, Collin J, Weishaar H, Gilmore AB. Is the increasing policy use of Impact Assessment in Europe likely to undermine efforts to achieve healthy public policy? Journal of Epidemiology and Community Health. 2010;64(6):478-87.

60. Smith KE, Fooks G, Collin J, Weishaar H, Mandal S, Gilmore AB. "Working the System''-British American Tobacco's Influence on the European Union Treaty and Its Implications for Policy: An Analysis of Internal Tobacco Industry Documents. Plos Medicine. 2010;7(1).

61. Smith KE, Fooks G, Gilmore AB, Collin J, Weishaar H. Corporate Coalitions and Policy Making in the European Union: How and Why British American Tobacco Promoted "Better Regulation". Journal of Health Politics Policy and Law. 2015;40(2):325-72.

62. Smoke Free Partnership. The origin of EU Better Regulation - the Disturbing Truth. Smoke Free Partnership; 2010.

63. Peeters S, Costa H, Stuckler D, McKee M, Gilmore AB. The revision of the 2014 European tobacco products directive: an analysis of the tobacco industry's attempts to 'break the health silo'. Tobacco Control. 2015;25:108-17.

64. Grundy Q, Bero L, Malone R. Interactions between Non-Physician Clinicians and Industry: A Systematic Review. Plos Medicine. 2013;10(11).

65. Spurling GK, Mansfield PR, Montgomery BD, Lexchin J, Doust J, Othman N, et al. Information from pharmaceutical companies and the quality, quantity and cost of physicians' prescribing: a systematic review. PLoS Medicine. 2010;7(10).

66. Sass JB. Industry efforts to weaken the EPA's classification of the carcinogenicity of 1,3-Butadiene. International Journal of Occupational and Environmental Health. 2005;11:378-83.

67. Pesticide ActionNetwork UK, Sustain, Lydgate E. Toxic Trade - how trade deals threaten to weaken UK pesticide standards. 2020.

68. Gasparyan AY, Ayvazyan L, Akazhanov NA, Kitas GD. Conflicts of interest in biomedical publications: considerations for authors, peer reviewers, and editors. Croatian Medical Journal. 2013;54(6):600-8.

1. The creation of favourable science is often managed by/outsourced to third parties, including: individual scientists (sometimes those whose views already align with industry), contract research organisations (often used by the pharmaceutical industry as ‘ghost-writing’ companies), university research groups, public relations companies, product defense firms, legal firms, scientific front groups, and at times, in collaboration with governments [↑](#footnote-ref-1)
2. Lawyers and/or PR firms have also been instrumental in the enactment of many other strategies including 3, 5-19. [↑](#footnote-ref-2)
3. For other ways of preventing potentially unfavourable science from reaching the public domain, see strategy 6 [↑](#footnote-ref-3)
4. When industry works to promote these links with external organisations, it can also serve to enhance industry’s perceived credibility (see strategy 18) [↑](#footnote-ref-4)
5. Created through strategies 1.1 and 3 [↑](#footnote-ref-5)
6. For more examples of attacks on scientists see Strategy 10 [↑](#footnote-ref-6)
7. By the late 1990s the industry’s “sound science” public relations campaign came to an end and “the tobacco industry then turned to advancing the ‘sound science’ concept through legislation” (Bero, 2013, p.162) – see strategy 16 [↑](#footnote-ref-7)
8. This strategy is also used as a way of harassing researchers with onerous data access requests and draining their resources – see strategy 10 [↑](#footnote-ref-8)
9. Such attacks on or misrepresentations of unfavourable science can take place within peer-reviewed contexts (such as through strategy 3) and beyond (such as through strategies 13-15), and are often conducted by third parties, see strategy 12. [↑](#footnote-ref-9)
10. Including using “good epidemiology” standards and “sound science” rhetoric (strategy 7) [↑](#footnote-ref-10)
11. Attacks on scientists are also used in attempts to prevent publication of science (strategy 6.2) and are also often combined with attacks on the scientists’ outputs (strategy 9). Here we focus on attacks which primarily target the researcher or scientific body [↑](#footnote-ref-11)
12. Often using PR and legal firms as intermediaries to facilitate the creation and funding of such groups and individuals [↑](#footnote-ref-12)
13. See strategies 8 and 16 [↑](#footnote-ref-13)
14. This is often done through third parties (see strategy 12) and through the media (strategy 15). When science is reproduced or packaged directly and overtly by industry, this can also function to enhance perceptions of industry’s scientific credibility (see strategy 18) [↑](#footnote-ref-14)
15. Scientific messages disseminated via the media include: promotion of favourable research (often that which is not peer-reviewed so has not been disseminated via scientific channels), attacks on unfavourable science and scientists, and commentary on scientific standards [↑](#footnote-ref-15)
16. In addition to disseminating scientific messages to policy decision-makers (strategy 12.2), industry actors have also gone further, with attempts to establish policymaking architecture and regulatory tools, in attempts to *guarantee* that industry-friendly scientific messages are heard [↑](#footnote-ref-16)
17. An example of misrepresentation of science as in strategy 9.4 [↑](#footnote-ref-17)
18. See strategies 1, 3, 5, 12, 13, and 14 for further strategies which contribute to industry’s presence in academic settings. [↑](#footnote-ref-18)
19. See strategy 12 for the messengers used, strategy 13 for how this science can be packaged, and strategy 14 for contexts in which dissemination of science takes place [↑](#footnote-ref-19)
